# Quantification of Pesticides and In Vitro Effects of Water-Soluble Fractions of Agricultural Soils in South Africa

**DOI:** 10.1007/s00244-025-01115-y

**Published:** 2025-02-15

**Authors:** Ilzé Engelbrecht, Suranie R. Horn, John P. Giesy, Rialet Pieters

**Affiliations:** 1 Unit for Environmental Sciences and Management, North-West University, Potchefstroom Campus, Private Bag X6001, Potchefstroom, 2520 South Africa; 2 Occupational Hygiene and Health Research Initiative, North-West University, Potchefstroom, 2520 South Africa; 3 Toxicology Program Faculty, Toxicology Centre, University of Saskatchewan, 44 Campus Drive, Saskatoon, SK S7N 5B3 Canada; 4 Department of Veterinary Biomedical Sciences, University of Saskatchewan, Saskatoon, SK S7N 5B4 Canada; 5 Department of Integrative Biology and Center for Integrative Toxicology, Michigan State University, East Lansing, MI 48824 USA; 6 Department of Environmental Science, Baylor University, One Bear Place #97266, Waco, TX 76798-7266 USA

## Abstract

Although agrochemicals protect crops and reduce losses, these chemicals can migrate to non-target environments via run-off and leaching following irrigation or heavy rainfall, where non-target organisms can be exposed to a mixture of water-soluble compounds. This study investigated whether the water-soluble fractions of selected agricultural soils from South Africa contain quantifiable concentrations of four commonly used pesticides, 2,4-dichlorophenoxyacetic acid (2,4-D), atrazine, dicamba and imidacloprid, and whether the aqueous extracts induce effects in vitro. Effects investigated included cytotoxicity using the MTT [3-(4,5-dimethylthiazol-2-yl)-2,5-diphenyltetrazolium bromide] cell viability assay; xenobiotic metabolism using the H4IIE-*luc* rat hepatoma cell line; and (anti-)androgenic and (anti-)oestrogenic effects were screened for with the human breast carcinoma cell lines MDA-kb2 and T47D-KB*luc*, respectively. Oxidative stress responses were measured in H4IIE-*luc* and human duodenum adenocarcinoma (HuTu-80) cells. All extracts of soil induced oxidative stress, while several samples caused moderate to severe cytotoxicity and/or anti-androgenic effects*.* The herbicide atrazine had the greatest frequency of detection (89%), followed by dicamba (84%), 2,4-D (74%) and imidacloprid (32%). Concentrations of atrazine [2.0 × 10^–1^ to 2.1 × 10^2^ ng/g, dry mass (dm)] and the neonicotinoid insecticide, imidacloprid (2.0 × 10^1^ to 9.7 × 10^1^ ng/g, dm), exceeded international soil quality guidelines. Overall, there was no observable trend between the biological effects and pesticides quantified. Nonetheless, the findings of this study show that agricultural soils in South Africa can elicit effects in vitro and contain quantifiable concentrations of polar pesticides. These agrochemicals might pose risks to the health of humans and the environment, but more assessment is necessary to quantify such potential effects.

**Supplementary Information:**

The online version contains supplementary material available at 10.1007/s00244-025-01115-y.

In sub-Saharan Africa, South Africa uses the greatest amounts of pesticides, with more than 3000 products registered for use (Dabrowski et al. 2014; Dabrowski 2015a). Although hydrophobic pesticides readily adsorb to soil particles, polar compounds are more mobile in soils and can leach by irrigation or rainfall into adjacent surface waters (Durães et al. 2018; Meftaul et al. 2019). This promotes the movement of polar compounds from soils to non-target aquatic environments, where if bioavailable, mixtures of these chemicals can pose risks to humans and wildlife (Kortenkamp 2008; Mandal et al. 2020). When xenobiotics are accumulated in cells (US NRC 2003), they can be stored, transformed, assimilated or degraded (Semple et al. 2004). However, during these processes, certain toxicological effects can occur, including cytotoxicity, endocrine disruption and oxidative stress (Jabłońska-Trypuć et al. 2017; Abdel-Halim and Osman 2020; Teng et al. 2020; Wang et al. 2021; Sule et al. 2022).

Cytotoxicity has no specified mechanism of action, but is used as an early warning for toxicity since it integrates responses elicited by chemicals present in environmental mixtures. On the other hand, endocrine disruption or oxidative stress is specific modes of action that can occur between chemicals like pesticides and cellular components (Escher et al. 2021). Endocrine disruption is any interference with the normal functioning of the endocrine system (Gore et al. 2015), including (i) binding to and activating hormone receptors (agonism), (ii) binding to and inhibiting hormone receptors (antagonism), (iii) alterations in hormone receptor expression, (iv) changes in hormone synthesis and (v) alterations in hormone transportation across cellular membranes (La Merrill et al. 2020). This is of concern because hormonal disruptions can cause reproductive dysfunction (Gore et al. 2015). Oxidative stress is the physiological imbalance between production and neutralisation of reactive oxygen species (ROS) by an organism’s antioxidant defence system (Burella et al. 2018; Jabłońska-Trypuć et al. 2017). Under normal physiological conditions, ROS play an essential role in cellular homeostasis by maintaining biological functions (Jabłońska-Trypuć et al. 2017; Pisoschi and Pop 2015; Ventura et al. 2015). However, excessive ROS production results in oxidative stress, which can cause damage to cell membranes and DNA. The antioxidant enzymes, superoxide dismutase (SOD) and catalase (CAT) prevent oxidative stress by converting superoxide radicals (O_2_^•–^) into hydrogen peroxide (H_2_O_2_) and O_2_, and catalysing the decomposition of subsequent H_2_O_2_ into water (H_2_O) and O_2_ (Chance 1948; McCord and Fridovich 1969; Kapoor et al. 2010).

Although previous studies have investigated effects of pesticide-active ingredients (Saggioro et al. 2019; Teng et al. 2020; Andrioli and Chaufan 2021), less attention has been paid to environmental mixtures (Prinsloo et al. 2013; Keita et al. 2021; Eze et al. 2023). Effect-based methods, such as in vitro bioassays, are used for the detection and quantification of effects elicited by chemical mixtures (Brack et al. 2019). These cell-based bioassays are relevant when screening for effects at the level of cellular response pathways such as those elicited by small concentrations of combined environmental chemicals (Jia et al. 2015; Horn et al. 2020; Pastorino et al. 2024). According to Dingemans et al. (2019), in vitro and small-scale in vivo methods are preferred over tests using whole organisms for rapid screening of toxicological effects in water. Although in vitro models cannot completely predict effects in whole organisms including wildlife and humans, cell lines retain some characteristics and functions of the organs from which they are derived (Nikolic et al. 2018; Mennillo et al. 2019). Consequently, mammalian cell lines, specifically human in vitro models, are relevant to human responses and allow predictions of human toxicological outcomes (Pastorino et al. 2024).

Some ecotoxicology studies use organic solvents as the extraction medium which force compounds not naturally bioavailable in the environment, into solution. However, this is not representative of environmental conditions. Due to the migration of polar agrochemicals through soil (Durães et al. 2018), there is a need to investigate the potential for adverse effects associated with the water-soluble (i.e. bioavailable) fraction of environmental matrices. Yet, in vitro bioassays alone cannot provide information about the identities of chemicals present in mixtures. Therefore, instrumental chemical analyses are used to complement cell-based methods (Brack et al. 2019). Such a combined approach provides a holistic overview of the impact of agriculture on non-target organisms in the environment.

This study determined whether four pesticides commonly used in the South African agriculture industry including, 2,4-dichlorophenoxyacetic acid (2,4-D), atrazine, dicamba and imidacloprid are present in agricultural soils from irrigated and rain-fed locations in South Africa using instrumental chemical analysis. These pesticides were selected because they (i) are registered for use in South Africa as an active ingredient in pesticide formulations for maize (Agri-Intel 2021); (ii) are listed under the top 25 ranked pesticides in the country based on quantity of use and hazard potential (2,4-D, atrazine and imidacloprid) (Dabrowski et al. 2014); and (iii) are moderately to highly soluble in water. Furthermore, the study investigated whether extractable, polar compounds present in the soils cause cytotoxicity, endocrine disruption, oxidative stress or activate xenobiotic metabolism by using relevant and standardised in vitro bioassays. This study was the first to show that the water-soluble fraction of agricultural soils from South Africa contains quantifiable concentrations of current-use pesticides and causes effects in vitro.

## Materials and Methods

### Chemicals and Media Constituents

*Standards:* 2,4-dichlorophenoxyacetic acid (2,4-D) (CAS 94–75-7), 17β-oestradiol (E_2_) (CAS 50–28-2), atrazine (CAS 1912–24-9), caffeine (CAS 58–08-2), dexamethasone (CAS 50–02-2), dicamba (CAS 1918–00-9), flutamide (CAS 13311–84-7), imidacloprid (CAS 138261–41-3) and testosterone (CAS 58–22-0) were purchased from Merck (Pty) Ltd (Modderfontein, South Africa), while 2,3,7,8-tetrachlorodibenzo-*para*-dioxin (TCDD) (CAS 1746–01-6) and fulvestrant (ICI) (CAS 129453–61-8) were purchased from Industrial Analytical (Pty) Ltd (Johannesburg, South Africa). *Sampling*: Ethanol (CAS 64–17-5) was purchased from Merck (Pty) Ltd (Modderfontein, South Africa). *Extraction:* Methanol (CAS 67–56-1) was purchased from Merck (Pty) Ltd (Modderfontein, South Africa). In vitro *bioassays:* 2’,7’-dichlorodihydrofluorescein diacetate (H_2_DCF-DA) (CAS 4091–99-0), 3-(4,5-dimethylthiazol-2-yl)-2,5-diphenyltetrazolium bromide (MTT) (CAS 298–93-1), 4-(2-hydroxyethyl)-1-piperazineethanesulphonic acid (HEPES) buffer (CAS 7365–45-9), bovine serum albumin (BSA) (CAS 9048–46-8), Bradford’s reagent, calcium chloride (CaCl_2_) (CAS 10043–52-4), D( +)-glucose (CAS 50–99-7), diethylenetriaminepentaacetic acid (DTPA) (CAS 67–43-6), dimethyl sulphoxide (DMSO) (CAS 67–68-5), Dulbecco’s modified Eagle’s medium (DMEM), Dulbecco’s phosphate-buffered saline (DPBS), ethanol (CAS 64–17-5), hexane (CAS 110–54-3), hydrogen peroxide (H_2_O_2_) (CAS 7722–84-1), Leibovitz (L-15) medium, magnesium sulphate heptahydrate (MgSO_4_.7H_2_O) (CAS 10034–99-8), methanol (CAS 67–56-1), potassium permanganate (KMnO_4_) (CAS 7722–64-7), pyrogallol (CAS 87–66-1), Roswell Park Memorial Institute (RPMI)-1640 medium, sodium bicarbonate (NaHCO_3_) (CAS 144–55-8), sodium pyruvate solution, sulphuric acid (H_2_SO_4_) (CAS 7664–93-9) and tris(hydroxymethyl)aminomethane) (tris-buffer) (CAS 77–86-1) were purchased from Merck (Pty) Ltd (Modderfontein, South Africa). Antibiotic–antimycotic mixture was obtained from Biowest (Nuaillé, France) and foetal bovine serum (FBS) from Capricorn Scientific GmbH (Ebsdorfergrund, Germany).

### Site Description

**Fig. 1:**
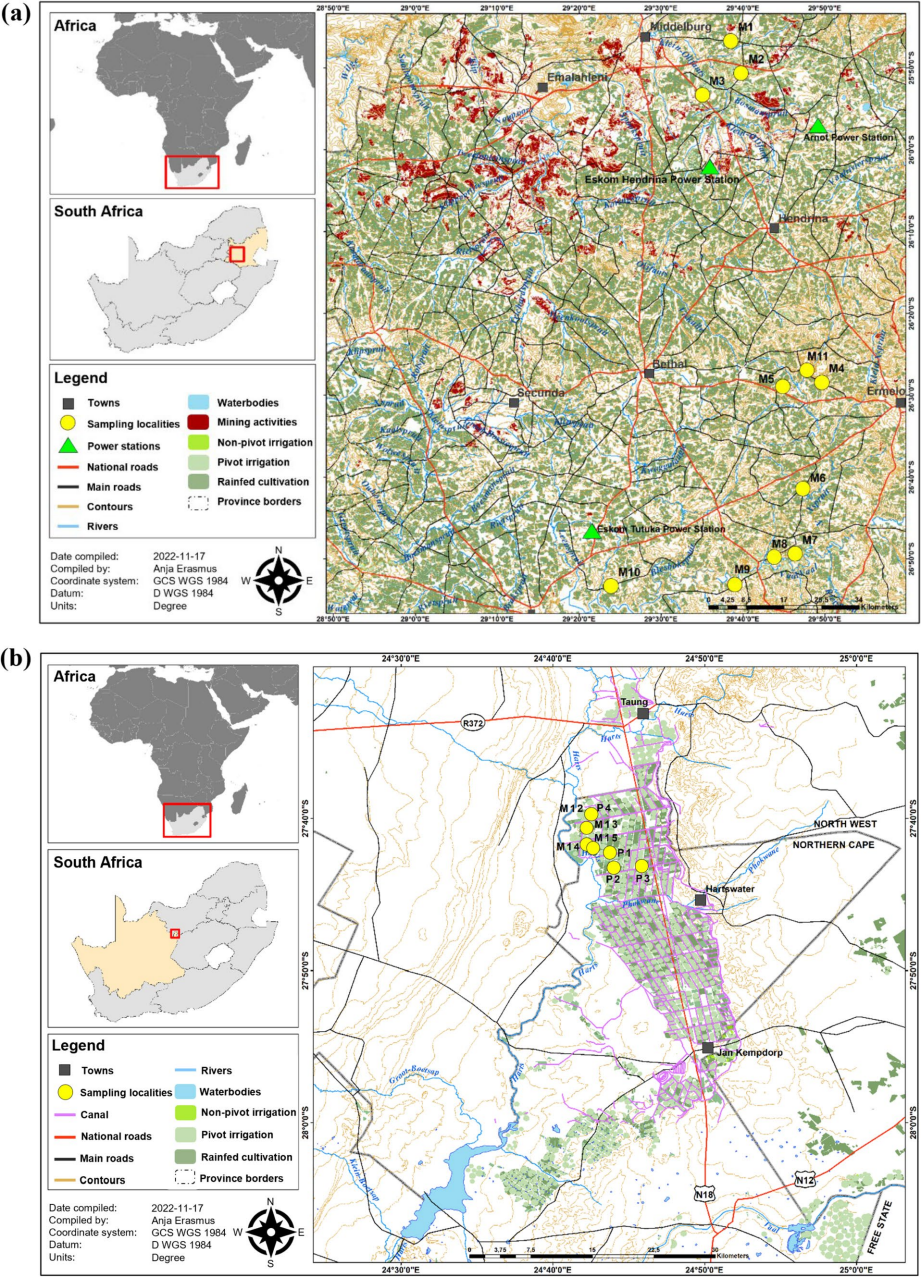
Sampling locations in **a** Mpumalanga province and **b** Vaalharts Valley in the north-east of the Northern Cape province. M1–15: Maize field 1–15; P1–4: pecan orchard 1–4

Two agricultural regions in South Africa that represent some of the largest inputs of pesticides, the Mpumalanga province (MP) and Vaalharts Valley (VH) in the Northern Cape province were selected as study areas (Dabrowski 2015b, 2015c). The MP is the largest maize-producing region in South Africa in terms of average yield (1000 t/ha) (Grain 2021), and herbicides are extensively applied. The MP has greater annual rainfall than the VH, and therefore, farmers mainly follow rain-fed cultivation practices. Since some of the sampling locations in the Highveld escarpment of the MP are located within the Upper Vaal River catchment and Klein Olifants River (Fig. 1a), applied pesticides can migrate to these water bodies following rainfall.

The VH is located between the Vaal and Harts Rivers, in the north-east of the Northern Cape province, bordering the North West province of South Africa (Fig. 1b). Maize is cultivated in the area, but in recent years, pecan nut farming has become more popular. Due to the drier climatic conditions, many of the farms in the area are irrigated with water from the Vaalharts Irrigation Scheme. In this scheme, water from a weir in the Lower Vaal River is diverted into a series of channels, siphons and pipes to irrigate crops (Verwey and Vermeulen 2011).

### Collection of Soil

Composite soil samples were collected in 250 mL ethanol-rinsed, high-density polyethylene bottles (Nalgene® Thermo Scientific™) from maize fields and pecan orchards in the MP and VH during the time of pesticide application in the maize-growing season (November–December 2020). The samples were transported back to the laboratory at 4 °C and stored at -20 °C until extraction of the polar compounds. All sampling equipment was rinsed with deionised water between sampling locations to avoid contamination.

Since the aim of the study was to screen for potential mixture effects of water-soluble agrochemicals and determine the presence of selected pesticides, a single sampling event was deemed sufficient for the scope of this survey, rather than conducting multiple sampling events for temporal comparison.

### Extraction of Polar Compounds from Soil for Biological Endpoint Determination

This study investigated potential adverse effects associated with water-soluble agrochemicals. To mimic the movement of agrochemicals from soils towards off-site locations after irrigation or rainfall events (Horn et al. 2020), compounds were extracted with ultrapure deionised water (18.2 MΩ-cm) of which the pH was adjusted based on the pH of the surface water and rainwater from the sampling areas (pH ~ 4 and pH ~ 7, for the MP and VH, respectively) (Conradie et al. 2016). Polar compounds were extracted by adding 20 mL of deionised water to 10 g of soil in a 50 mL centrifuge tube and shaking the mixture at 150 rpm for 1 h on a mechanical shaker. This was followed by centrifugation at 3000 g for 20 min (Horn et al. 2020). The process was repeated twice, and the supernatants were pooled and used to prepare the samples for the in vitro bioassays.

### In vitro Bioassays

#### Cell Culture Maintenance

The HuTu-80 (ATCC® HT4-40™) and T47D-KB*luc* (ATCC® CRL-2865™) cell lines were acquired from the American Type Culture Collection (Virginia, USA), while the H4IIE-*luc* and MDA-kb2 cells were gifted by the University of Saskatchewan (Saskatchewan, Canada). Before the experiments, each cell line was routinely cultured in their respective nutrient medium under sterile conditions in tissue culture dishes in humidified air supplemented with 5% carbon dioxide (CO_2_) (except the MDA-kb2 cells) at 37 °C (Table 1).

**Table 1:** Growth conditions of each specific cell line

Cell line	Nuclear receptor	Nutrient medium and constituents	pH	Seeding density (cells/mL)	Solvent control	Reference compound
*Routine cell culture maintenance*						
HuTu-80 (Human duodenum)	–	DMEM with low glucose and without phenol red, 3.7 g/L NaHCO_3_, 10% FBS, 1% antibiotic–antimycotic mixture*	7.1	–	–	–
H4IIE-*luc* (Rat hepatoma)	AhR	DMEM with low glucose and without phenol red, 3.7 g/L NaHCO_3_, 10% FBS	7.1	–	–	–
MDA-kb2 (Human breast carcinoma)	AR and GR	Leibovitz (L-15) medium containing L-glutamine, 10% FBS, 1% antibiotic–antimycotic mixture^†^	7.3	–	–	–
T47D-KB*luc* (Human breast carcinoma)	ER	RPMI-1640 medium, 2.5 g/L D( +)-glucose, 1.5 g/L NaHCO_3_, 100 mM sodium pyruvate solution, 1 M HEPES buffer, 10% FBS or 10% CDT FBS (three and seven days before the assessment of ER agonism and antagonism, respectively)	7.3	–	–	–
In vitro *bioassays*						
HuTu-80 (Human duodenum)	–	Routine cell culture medium (see the first half of the table)	7.1	80 000	–	–
H4IIE-*luc* (Rat hepatoma)	AhR↑	Routine cell culture medium but 10% CDT FBS^‡^ replaced FBS	7.1	80 000	Hexane	TCDD
MDA-kb2 (Human breast carcinoma)	AR↑	Routine cell culture medium but 10% CDT FBS^‡^ replaced FBS	7.3	120 000	Methanol	Testosterone
	AR↓	Routine cell culture medium but 10% CDT FBS^‡^ replaced FBS, 0.283 ng/mL testosterone				Flutamide
	GR↑	Routine cell culture medium but 10% CDT FBS^‡^ replaced FBS, 0.5942 µg/mL flutamide				Dexamethasone
T47D-KB*luc* (Human breast carcinoma)	ER↑	Routine cell culture medium but 10% HyClone™ CDT FBS^§^ replaced FBS used in routine culture medium, 0.04 ng/mL ICI	7.3	120 000	Methanol	E_2_
	ER↓	Routine cell culture medium but 10% HyClone™ CDT FBS^§^ replaced FBS used in routine culture medium, 5.4 pg/mL E_2_				ICI

AhR: Aryl hydrocarbon receptor; AR: androgen receptor; CDT: charcoal dextran-treated; CO_2_: carbon dioxide; DMEM: Dulbecco’s modified Eagle’s medium; E_2_: 17β-oestradiol; ER: oestrogen receptor; FBS: foetal bovine serum; HEPES: 4-(2-hydroxyethyl)-1-piperazineethanesulphonic acid; ICI: fulvestrant; NaHCO_3_: sodium bicarbonate; RPMI-1640: Roswell Park Memorial Institute-1640; TCDD: 2,3,7,8-tetrachlorodibenzo-*para*-dioxin; −not applicable; ↑ agonism; ↓ antagonism. *The HuTu-80 cell line originates from the human gastrointestinal tract that is naturally colonised by bacteria. Therefore, the routine culture of the cells under laboratory conditions required the use of antibiotics. The cell line also does not contain reporter genes that are sensitive to interference by antibiotics. ^†^MDA-kb2 cells were routinely cultured with a 1% antibiotic–antimycotic mixture to combat persistent bacterial infection. Although the use of antibiotics can interfere with the outcome of reporter gene bioassays, previous experiments in our laboratory showed that the use of antibiotics did not affect binding to the AR; ^‡^CDT FBS (i.e. from which hormones had been removed) was only used for the reporter gene bioassays (hormone-sensitive), and hormone-rich FBS was used for all oxidative stress bioassays (not hormone-sensitive assays); ^§^commercially available FBS from which hormones had been removed

### Sample Preparation

During assays of cytotoxicity, reporter gene and oxidative stress bioassays, cells were exposed to the bioavailable fraction of the samples by using extracts of the soil to prepare nutrient media. The pH ~ 4 aqueous extracts were used for the MP samples, while the pH ~ 7 extracts were used for the VH samples. This was done by adding 20 mL of supernatant from the extraction procedure to a centrifuge tube containing the cells’ respective powdered nutrient media and constituents as listed in Table 1 (Horn et al. 2020). The pH of the media was adjusted, and the samples were sterile filtered using a 22 μm syringe filter (Escher et al. 2021) and serially diluted to obtain the following exposure concentrations: 1, 3, 9, 28, 83 and 250 mg soil equivalents/mL.

### Cell Viability Assay (MTT)

The MTT [3-(4,5-dimethylthiazol-2-yl)-2,5-diphenyltetrazolium bromide] cell viability assay was used to determine the cytotoxic potential of extracts. Additionally, separate MTT cell viability assays were conducted in parallel with the luminescence reporter gene bioassays for quality control, i.e. to prevent false negative luminescence reporter gene assay results as cytotoxicity can mask the effects of receptor-mediated bioassays (Escher et al. 2021). This viability assay measures the ability of the mitochondria in living cells to metabolise MTT through the activity of their enzymes (Mosmann 1983). The results were expressed as a percentage (%) of the solvent control (SC) which represents 100% viable cells. All exposures were done in triplicate.

### Reporter Gene Bioassays

Reporter gene bioassays were used to determine whether samples elicit receptor-mediated effects. During this assay, ligands bind to nuclear receptors, resulting in the expression of transactivation, reporter gene (*luc*) and synthesis of luciferase. Luciferase activity is quantified luminometrically, and the amount of light emitted (relative light units, RLUs) is proportional to the number of ligands present in the samples that bind to the receptor (Denison et al. 2004).

#### Xenobiotic Metabolism

The H4IIE-*luc* cells show aryl hydrocarbon receptor (AhR)-controlled luciferase expression and were used to investigate whether the samples contain AhR ligands that activate xenobiotic metabolism (Aarts et al. 1993) (see Supplementary Information for a detailed description of the method).

#### (Anti-)Androgenic Activity

The MDA-kb2 cell line expresses both the androgen (AR) and glucocorticoid receptor (GR) (Wilson et al. 2002) and was used to determine the (anti-)androgenic activity of the samples (see Supplementary Information for a detailed description of the method).

#### (Anti-)Oestrogenic Activity

T47D-KB*luc* cells naturally express the α- and β-ER, with an oestrogen response element promoter–luciferase reporter gene construct (Wilson et al. 2004). The method described here is adapted because of an unknown source of oestrogen contamination in our tissue culture laboratory. This adaptation would still allow comparison between samples in this study, but not to other studies which followed the usual protocol.

Three days before the assessment of ER agonism, cells were cultured in a nutrient medium stripped of hormones by replacing the 10% FBS with 10% charcoal dextran-treated (CDT) FBS (Wilson et al. 2004) (Table 1). At the start of the assay, cells were seeded in a medium from which hormones had been removed, in 96-well microplates (Table 1). After 24 h of incubation, cells were exposed to the samples. To minimise the effect of the additional oestrogen and override any oestrogenic contamination from interfering with the assessment of ER agonism, the samples contained a background concentration of the ER antagonist, ICI (0.04 ng/mL) (Table 1). A background concentration of ICI ensured competitive binding for the ER between the ICI and any potential ER agonists present in the samples. 17β-oestradiol (E_2_) diluted in the hormone-stripped medium (0.02, 0.11, 0.39, 1.36, 2.72 and 6.81 pg/mL) was the reference compound. A nutrient medium was used to prepare the E_2_ concentrations to minimise any effect on the ER. Ethanol for example has been found to stimulate ER overexpression (Chen et al. 2009). After 24 h of exposure, luciferase activity was determined.

For the evaluation of ER antagonism, cells were cultured in the hormone-stripped medium for seven days before use in in vitro bioassays (Table 1). For ER antagonism, cells were seeded in the hormone-stripped medium in 96-well microplates (Table 1). After 24 h of adherent growth, the cells were exposed to the samples. The samples contained a background concentration of E_2_ (5.4 pg/mL) which caused 80% activation of the ER (Escher et al. 2021). ICI serially diluted in ethanol (0.02, 0.05, 0.16, 0.47, 1.41 and 4.22 ng/mL) was the reference compound. After 24 h of exposure, luciferase activity was determined as previously described for the other reporter gene bioassays in Supplementary Information.

During the reporter gene bioassays, all exposures were done in triplicate. Cells were exposed to media containing various amounts of soil extracts instead of culture medium, while cells were directly dosed with reference compounds in nutrient media. During data calculations, the reference compound responses were plotted on a logarithmic scale to obtain a sigmoidal concentration–response curve (Escher et al. 2021). Sample responses were compared to the reference compounds and expressed in terms of %TCDD Max, %Testosterone Max %Flutamide Max, %Dexamethasone Max, %E_2_ Max and %ICI Max. However, since sample responses did not resemble a classical concentration–response curve, bioanalytical equivalents could not be calculated. Rather, a fold change (FC) was calculated by dividing the mean RLUs of the samples at 250 mg/mL by the mean RLUs of the SC (Aït-Aïssa et al. 2010; Wilson et al. 2002). An FC > 1 or FC < 1 was considered indicative of receptor agonism or antagonism, respectively.

### Measurement of Oxidative Stress

Potencies of extracts to cause oxidative stress were evaluated in human duodenum adenocarcinoma (HuTu-80) and rat hepatoma (H4IIE-*luc*) cell lines. These cell lines were selected since they originate from detoxification organs and consequently retain an array of organ functions including anti-oxidative capabilities (Röhrdanz et al. 2002; Pereira Moreira et al. 2009; Mennillo et al. 2019). The gastrointestinal tract (GIT) is the most common exposure route for xenobiotics, with parent compounds and metabolites absorbed through the GIT (Ryu et al. 2021). After the absorption of xenobiotics in the GIT, the liver is responsible for xenobiotic metabolism, including phase I and II biotransformation reactions which increase ROS production (Gu and Manautou 2012; Ray et al. 2012; He et al. 2017). Consequently, the HuTu-80 and H4IIE-*luc* cells were considered suitable models for studying oxidative stress responses elicited by environmental samples.

#### ROS Generation

Reactive oxygen species production was measured using H_2_DCF-DA according to the methods described by Wang and Joseph (1999), Halliwell and Whitemann (2004) and Engelbrecht et al. (2024). This fluorogenic dye is hydrolysed intra-cellularly to non-fluorescent 2’,7’-dichlorodihydrofluorescein where it reacts with ROS, generating fluorescent 2’,7’-dichlorofluorescein (Wang and Joseph 1999) (see Supplementary Information for a detailed description of the method).

#### Protein Determination and Antioxidant Enzyme Assays

Protein content was used as a measure to normalise all antioxidant enzyme assay data. The total protein concentration was determined according to Bradford (1976). Superoxide dismutase content was determined using the pyrogallol autoxidation method described by Marklund and Marklund (1974), Del Maestro and McDonald (1987), and Engelbrecht et al. (2023). Catalase activity was measured as a function of the enzyme-catalysed decomposition of H_2_O_2_. The amount of H_2_O_2_ remaining after CAT activity was determined by titration with an excess of a strong oxidising reagent, KMnO_4_, as described by Cohen et al. (1970) and Engelbrecht et al. (2023) (see Supplementary Information for a detailed description of the method).

### Data Analyses

Results for cell viability were interpreted according to the ISO 10993–5 guidelines as follows: non-toxic (> 80%); weakly cytotoxic (60–80%); moderately cytotoxic (40–60%); and strongly cytotoxic (< 40%) (ISO 2009). This standard was previously used to interpret results of cell viability in a HaCaT keratinocyte cell line by López-García et al. (2014) and was therefore deemed suitable. For all in vitro bioassays, Microsoft Excel and the SPSS statistical package version 24 (IBM) were used to determine statistically significant differences in responses between exposed and control cells. The Kolmogorov–Smirnov test was used to determine normality, but since the data were not normally distributed, the nonparametric Mann–Whitney *U* test was used for statistical analysis with *p* ≤ 0.05 and *p* ≤ 0.01 where applicable. Cohen’s d-value was determined for all oxidative stress responses to determine practical significance (d ≥ 0.8) (Ellis and Steyn 2003). All data are presented as mean ± standard deviation.

### Instrumental Chemical Analysis

#### Extraction for Instrumental Analysis

The soil samples were dried, ground and sieved. The target compounds were extracted from 80 g soil (spiked with 0.005 µg/mL caffeine as internal standard) (Meng 2008) with methanol using accelerated solvent extraction (DIONEX ASE 100®). Extraction was performed as described by Vonberg et al. (2014) with a few modifications (see Supplementary Information for a detailed description of the method).

Before the extraction of pesticides with methanol, polar compounds were extracted from soils by use of pH-adjusted deionised water (as for the in vitro bioassays) and chemically screened for the presence of pesticides. However, no pesticides were detected most likely since water was the extraction medium and these samples had not been concentrated. The feasibility of water as the extraction medium depends on the target compound and downstream use of the extracts. In vitro bioassays are highly sensitive and can quantify the effects of environmentally low concentrations. Therefore, unconcentrated water extracts could be used in the bioassays. Contrary to this, environmental concentrations of chemicals often fall well below the limit of detection of analytical instruments (Neale et al. 2020). Therefore, methanol was used as the extraction solvent to specifically target polar compounds because it shows better recovery of pesticides from soils for analytical applications (Atalay and Hwang 1999).

### Quality Control and Assurance

The extraction and chemical analysis were validated for matrix effects, linearity, precision and accuracy. An external matrix-matched calibration curve (spiked post-extraction) accounted for matrix effects (Wu et al. 2022) and was prepared by extracting 80 g of blank soil and spiking it with pesticide standards and caffeine (i.e., internal standard) post-extraction. The pesticide standard concentrations were prepared to deliver a linear range of the target compounds (Table 2). Coefficients of determination (R^2^) (as close to one as possible) were used to assess the linearity of the matrix-matched calibration curve (Miller and Miller 2018). Quality control (QC) samples were spiked with low, medium, and high concentrations of the target pesticides (Table 3) to determine the recovery (%) of the ASE extraction method. Accuracy was presented as a percentage of target compounds recovered from the QC sample in three concentrations (low, medium, and high). Precision was expressed as relative standard deviation (RSD). For intra-day precision, six replicates of a QC sample from the same day were injected. For inter-day precision, a QC sample was injected six times a day, over three days (Wu et al. 2022). The limit of detection (LOD) and limit of quantification (LOQ) were also determined (Schoeman et al. 2015). Matrix-matched calibrations and linear regression were used to calculate the pesticide concentrations in the QC samples. Experimental blanks that went through the same extraction process were also included.

**Table 2:** Concentrations (µg/mL) of target pesticides used for the matrix-matched calibration curve

Nr	2,4-D	Atrazine	Dicamba	Imidacloprid
1	0.000	0.000	0.000	0.000
2	0.050	0.005	0.750	0.500
3	0.850	0.008	1.200	1.200
4	0.250	0.013	2.000	2.500
5	0.500	0.030	3.800	4.000
6	0.800	0.050	6.000	6.000
7	1.300	0.075	10.500	8.000
8	2.000	0.100	15.000	10.000

^2^^,4-D: 2,4-Dichlorophenoxyacetic acid^

**Table 3:** Concentrations (µg/mL) of target pesticides in the quality control samples

Concentration	2,4-D	Atrazine	Dicamba	Imidacloprid
Low	0.085	0.008	1.200	1.200
Medium	0.500	0.030	3.800	4.000
High	1.300	0.075	10.500	8.000

^2^^,4-D: 2,4-Dichlorophenoxyacetic acid^

### Quantification of Target Pesticides

For details of this section, consult Supplementary Information. Chemical analysis of the extracts was adapted from a method by Meng (2008). Separation of the compounds was done with a ZORBAX Eclipse Plus C18 column on an ultrahigh-performance liquid chromatograph (UHPLC) and detected with a quadrupole time-of-flight mass spectrometer (QTOF/MS). Both positive and negative electron spray ionisation was used. Mobile phase A consisted of ultrapure water and mobile phase B was acetonitrile—both contained 0.1% formic acid as well (Supplementary Information, Table S1). For accurate mass references, a solution containing masses 121.050873 [M^+^H]^+^ and 922.009798 [M^+^H]^+^ was constantly infused into the QTOF/MS. Confirmation of target compounds was based on the specific retention time and accurate masses (m/z) (Table 4).

**Table 4:** Ion source, accurate mass and retention time of target compounds

Compound	Ion source	Accurate mass (m/z)	Retention time (min)
2,4-D product	Positive	218.9631	6.00
Atrazine	Positive	216.1057	8.80
Caffeine	Positive	195.0901	1.00
Dicamba product	Negative	218.9636	4.50
Imidacloprid	Positive	256.0622	2.50

^2^^,4-D: 2,4-Dichlorophenoxyacetic acid^

### Data Calculations

The data obtained after the chromatographic analysis of the samples by the UHPLC-QTOF/MS were used to quantify the target compounds (i.e., 2,4-D, atrazine, dicamba, and imidacloprid). Agilent’s MassHunter Data Acquisition (version 10.1 B.10.1.733.0) and Quantitative Analysis for QTOF (version 10.0 B.10.0.10305.0) were used for data processing. Pesticide concentrations were calculated based on matrix-matched calibrations run at known concentrations to obtain their corresponding peak area (abundance) responses. The sample extracts of unknown concentrations were analysed in triplicate, and based on the following linear regression formula, their relative responses were used to calculate their concentrations (x) (Eq. 1).

1 $${\text{x }} = \, \left( {\left( {{\text{native}}/{\text{IS}}} \right) \, {-}{\text{ c}}/{\text{m}}} \right) \, \times {\text{ IS concentration}}$$

with x = calculated concentration (µg/mL) of the target compound; native = target compound relative response (abundance); IS = internal standard (caffeine) relative response (abundance); c = y-intercept; m = slope; and IS concentration = internal standard concentration.

The calculated concentrations (µg/mL) were converted to µg/g, dry mass (dm) soil, and expressed as the mean ± standard deviation.

## Results

### Cytotoxicity

**Fig. 2:**
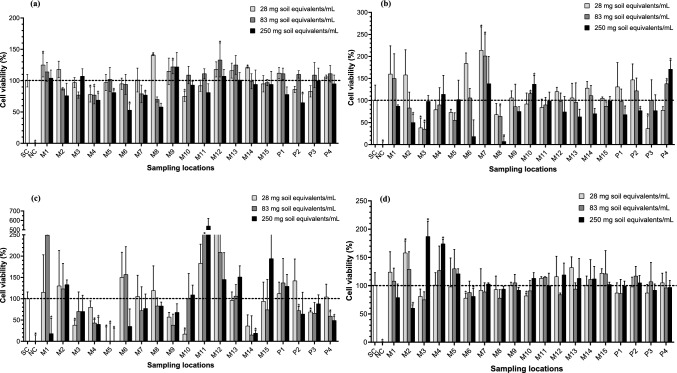
Cell viability (%) of the **a** HuTu-80, **b** H4IIE-*luc*, **c** MDA-kb2 (AR agonism) and **d** T47D-KB*luc* (ER agonism) cells after exposure to samples. Results are expressed in terms of the solvent control (SC) that represents 100% viability (dashed line). All data are presented as mean ± standard deviation. *Statistically significant compared to SC (*p* ≤ 0.05); M1–15: maize field 1–15; NC: negative control; P1–4: pecan orchard 1–4

Overall, cells exposed to an increasing concentration of samples showed weak to no cytotoxicity. However, there were a few exceptions. One undiluted MP sample, M6, was moderately cytotoxic (*p* ≤ 0.05) towards the HuTu-80 cells (Fig. 2a). On the other hand, one VH sample (P3) and three MP samples (M2, M3 and M8) caused cytotoxicity in the H4IIE-*luc* cells, with the greatest concentration (250 mg soil equivalents/mL) of M8 reducing cell viability with 93% (Fig. 2b). In the MDA-kb2 cells, strong cytotoxicity (*p* ≤ 0.05) was observed for several of the MP and VH samples including M1, M3, M4, M10, M14 and P4, while all the exposure concentrations of M5 were cytotoxic (Fig. 2c). Most samples did not cause cytotoxicity in the T47D-KB*luc* cell line, with relatively great cell viability (> 80%) reported overall. Only one MP sample, M2, was weakly cytotoxic at 250 mg soil equivalents/mL (Fig. 2d).

The least concentration of 28 mg soil equivalents/mL of some samples caused statistically significant (*p* ≤ 0.05) cytotoxicity in the H4IIE-*luc* (M3 and P3; Fig. 2b) and MDA-kb2 cells (M3 and M10; Fig. 2c), while the greatest concentration caused no cytotoxicity.

In contrast, several samples increased (*p* ≤ 0.05) cell viability to above 100% compared to the SC in the HuTu-80 (M1, M8, M9, M12 and M14; Fig. 2a), H4IIE-*luc* (M7, M10 and P4; Fig. 2b), MDA-kb2 (M11; Fig. 2c) and T47D-KB*luc* (M2–M4; Fig. 2d) cells. In general, 83 mg soil equivalents/mL was the highest non-cytotoxic concentration for all samples and was consequently used for the assessment of oxidative stress responses.

### Xenobiotic Metabolism

The dose–response curve for TCDD is shown in Supplementary Information (Fig. S1). Compared to the reference compound, none of the samples caused AhR agonism with all %TCDD Max values < 20% (Supplementary Information, Table S2).

### Endocrine Disruption

**Fig. 3:**
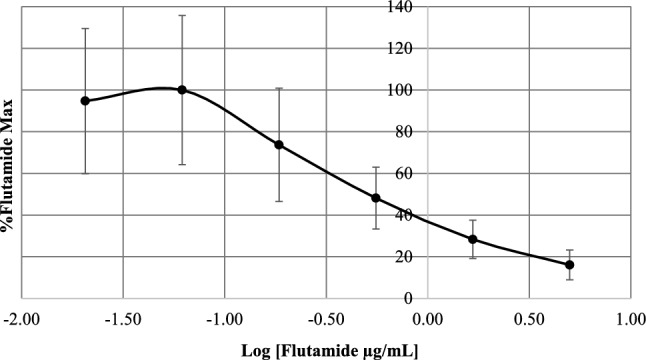
Dose–response curve for the androgen receptor antagonism reference compound, flutamide, during the MDA-kb2 bioassay. Luciferase activity is expressed as %Flutamide Max against the logarithmically transformed flutamide exposure concentrations (0.02, 0.06, 0.19, 0.56, 1.67, and 5.0 µg/mL). Error bars indicate the standard deviation

**Fig. 4:**
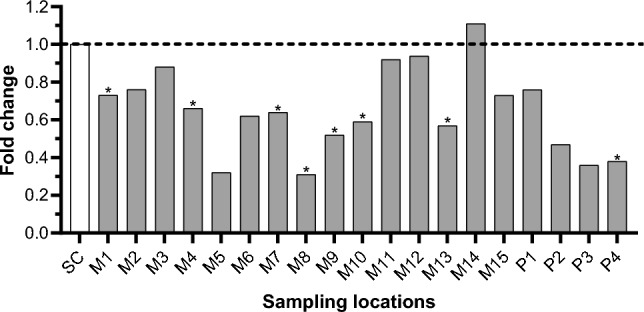
Fold change values for anti-androgenic activity at 250 mg soil equivalents/mL. Sample responses were compared to the solvent control (SC) (i.e., methanol) with FC = 1 (dashed line). *Statistically significant (*p* ≤ 0.05) compared to SC; M1–15: maize field 1–15; P1–4: pecan orchard 1–4

The dose–response curve for testosterone is shown in Supplementary Information (Fig. S2). However, during the assessment of AR agonism, all samples had %Testosterone Max values < 20%, which indicated no androgenic effects (Supplementary Information, Table S3). For some samples, this could be attributed to reported cytotoxicity (Fig. 2c). Since no receptor agonism was observed, a second bioassay was not performed, and GR agonistic activity was not further investigated. Alternatively, several MP and VH samples exhibited potential anti-androgenic effects. Consequently, viable cells validate that low %Flutamide Max values are indicative of receptor antagonism and not due to dead cells. However, these samples did not exhibit a classical concentration–response curve as was obtained for the reference compound, flutamide (Fig. 3), and consequently, only FC values could be calculated (Fig. 4). Most samples had an FC < 1, but the responses were only statistically significant (*p* ≤ 0.05) for M1, M4, M7–M10, M13, and P4 (Fig. 4). This suggests potential AR antagonism. In contrast to the results for cell viability during the assessment of AR agonism (Fig. 2c), none of these samples (M1, M4, M7–M10, M13) exhibited moderate to severe cytotoxic effects in the MTT assay run in parallel with the AR antagonism bioassay (Supplementary Information, Fig. S3).

During the evaluation of ER agonism, none of the samples showed any oestrogenic effects (Supplementary Information, Table S4). This was also the case for ER antagonism with none of the samples exhibiting anti-oestrogenic effects (Supplementary Information, Table S5). However, to validate the adapted methods followed, the dose–response curves for E_2_ and ICI are shown in Supplementary Information (Fig. S4 and S5).

### Oxidative Stress

#### Reactive Oxygen Species

Exposure to almost half of the MP samples (M1–M5) significantly increased (*p* ≤ 0.05; d ≥ 0.8) fluorescence intensity in HuTu-80 cells, while the other half (M6–M11) caused a reduction compared to the control (Tables 5 and S6). Alternatively, only one VH sample (P1) increased ROS levels and another (P3) decreased. Overall, M5 produced the greatest amount of ROS, producing twice as much fluorescence as the control, while M7 was responsible for a fourfold reduction (*p* ≤ 0.01; d ≥ 0.8) (Tables 5 and S6). In H4IIE-*luc* cells, three MP samples (M1, M5 and M7) significantly increased ROS levels, whereas M4, M8 and M9 reduced the amount of fluorescence emitted (Tables 5 and S6). A similar trend was seen for the VH samples with M12, M13 and M14 inducing the generation of ROS, while M15, P3 and P4 caused a decrease (*p* ≤ 0.05; d ≥ 0.8).

**Table 5:** Statistically significant oxidative stress responses in the HuTu-80 and H4IIE-*luc* cells after exposure to samples (83 mg soil equivalents/mL) for 24 and 72 h, respectively

Sampling location	HuTu-80			H4IIE-*luc*		
	ROS	SOD	CAT	ROS	SOD	CAT
*Mpumalanga province*						
M1	↑	↑	–	↑	–	–
M2	↑	–	–	–	↑	–
M3	↑	↑	–	–	–	–
M4	↑	–	–	↓	↑	–
M5	↑	–	–	↑	↑	–
M6	↓	–	–	–	↑	–
M7	↓	↑	↑	↑	↑	–
M8	↓	↓	↑	↓	↑	–
M9	↓	–	↓	↓	–	↑
M10	↓	↑	↑	–	–	–
M11	↓	↑	↑	–	↓	–
*Vaalharts Valley, Northern Cape province*						
M12	–	↓	↑	↑	–	↑
M13	–	↓	↑	↑	–	–
M14	–	↓	↑	↑	–	↑
M15	–	↓	↑	↓	–	–
P1	↑	↓	–	–	–	↑
P2	–	↓	↑	↑	–	–
P3	↓	↓	↑	↓	–	↑
P4	–	↓	↑	↓	–	–

↑ Increase; ↓ decrease; −no significant response; CAT: catalase; M1–15: maize field 1–15; P1–4: pecan orchard 1–4; ROS: reactive oxygen species; SOD: superoxide dismutase

### Antioxidant Enzymes

Exposure to the samples significantly affected antioxidant enzyme levels. Five MP samples (M1, M3, M7, M10 and M11) increased SOD content in HuTu-80 cells, while M8 reduced (*p* ≤ 0.01; d ≥ 0.8) the enzyme’s content 12-fold to 10 ng SOD/mg protein (Tables 5 and S6). All VH samples decreased SOD content. Most of the MP samples significantly increased SOD content in the H4IIE-5*luc* cells (Tables 5 and S6). Only M11 reduced (*p* ≤ 0.05; d ≥ 0.8) the enzyme’s concentration. Contrary to the HuTu-80 cells, no significant effect on SOD content was observed in the H4IIE-*luc* cells following exposure to the VH samples (Tables 5 and S6).

In terms of CAT, four MP samples (M7, M8, M10 and M11) significantly increased the enzymatic activity in HuTu-80 cells, while only M9 caused a decrease over the control (Tables 5 and S6). All VH samples increased (*p* ≤ 0.05; d ≥ 0.8) CAT activity, except P1. In general, MP samples did not affect CAT activity in the H4IIE-*luc* cells. However, a few VH samples (M12, M14, P1 and P3) increased CAT activity (Tables 5 and S6).

### Chemical Analysis

#### Method Validation

To measure the concentrations of 2,4-D, atrazine, dicamba and imidacloprid in agricultural soils from South Africa, an analytical method was developed and validated. The LODs and LOQs of the pesticides are reported (Table 6). The standard curves of all four pesticides had an R^2^ ≥ 0.995. The linearity, precision and accuracy were also acceptable. The results of the laboratory blanks were < LOQ, confirming that sample processing did not introduce any pesticides. The pesticide calibration curve ranges were as follows: 2,4-D, atrazine, dicamba and imidacloprid were: 0.0–2.0 (2,4-D), 0.0–0.1 (atrazine), 0.0–10.5 (dicamba) and 0.0–10.0 µg/mL (imidacloprid) (Table 6).

**Table 6:** Results of quality control and assurance for the instrumental chemical analyses

Pesticide	Calibration curve concentration range	Linearity	LOD	LOQ	Inter-day precision (%RSD)			Intra-day precision (%RSD)	Accuracy (%)
	µg/mL	R^2^	µg/mL	µg/mL	Day 1	Day 2	Day 3	< 15%	(Recovery)
2,4-D	0.0, 0.25, 0.5, 1.3, 2.0	0.995	0.197	0.655					36
Low					8.73	6.25	8.41	10.52	
High					2.56	2.24	5.75	5.17	
Medium					5.76	2.24	3.26	5.17	
Atrazine	0.0, 0.005, 0.0125, 0.05, 0.1	0.999	0.005	0.016					42
Low					10.62	18.37	12.70	20.22	
Medium					7.71	7.22	6.45	12.89	
High					3.26	5.50	8.34	14.70	
Dicamba	0.0, 2.0, 3.8, 6.0, 10.5	0.999	0.258	0.861					22
Low					22.17	13.40	22.68	24.00	
Medium					17.85	14.92	14.32	18.21	
High					21.37	9.92	12.08	17.57	
Imidacloprid	0.0, 0.5, 1.2, 6.0, 10.0	0.998	0.622	2.074					32
Low					11.62	14.69	3.91	14.60	
Medium					4.97	2.79	6.03	15.67	
High					1.99	3.89	3.39	15.24	

2,4-D: 2,4-Dichlorophenoxyacetic acid; LOD: limit of detection; LOQ: limit of quantification; R^2^: coefficient of determination; RSD: relative standard deviation

Low (< 50%) pesticide recoveries were obtained in this study (Table 6). However, for 2,4-D and atrazine, the %recoveries were similar to previous reports for polar pesticides from soil. The recovery of 2,4-D (36%) was in the range (25–67%) of that reported by Voos et al. (1994), while the recovery of atrazine (42%) was comparable to findings by Guzzella and Pozzoni (1999) who reported 47% recovery after spiking soil with 10 ng/g atrazine. In contrast to the current study, greater recoveries have been reported for dicamba (65–137%; Voos et al. 1994) and imidacloprid (77.1–94.8%; Bonmatin et al. 2003; 82.6–109%; Moghaddam et al. 2012; 78.8%; Schaafsma et al. 2015). It is, therefore, possible that the chosen extraction method might have contributed to the overall low recoveries. No correction factor was applied to recalculate the target compounds’ concentration, and since the recoveries are low, the actual concentrations are expected to be higher than reported (Table 6).

Greater recoveries of polar pesticides have been reported for use of QuEChERS (quick, easy, cheap, effective, rugged and safe) extraction kits as opposed to ASE (Bonmatin et al. 2003; Moghaddam et al. 2012; Schaafsma et al. 2015). In the present study, the extraction method used was not optimised for each target compound since the aim was to obtain several pesticides by using one extraction method. Moreover, the complexity of the matrix can affect recovery. According to Guzzella and Pozzoni (1999), the recovery of compounds from topsoil (0–25 cm) samples is usually lower compared to deeper soil samples because of the presence of humic acids, water, and other organic pollutants in the upper soil layer.

#### Quantification of Target Pesticides

All four target pesticides (2,4-D, atrazine, dicamba and imidacloprid) were detected at quantifiable concentrations in the soil samples (Table 7). Concentrations of pesticides ranged from 4.5 × 10^0^–8.3 × 10^2^ ng/g, dm for 2,4-D, 2.0 × 10^–1^–2.1 × 10^2^ ng/g, dm for atrazine, 4.9 × 10^1^–8.1 × 10^3^ ng/g, dm for dicamba, and 2.0 × 10^1^–9.7 × 10^1^ ng/g, dm for imidacloprid. Frequencies of detection were: atrazine (89%) > dicamba (84%) > 2,4-D (74%) > imidacloprid (32%) (Table 7). The greatest concentration quantified was dicamba (8.1 × 10^3^ ng/g, dm) in M11, and the least was atrazine (2.0 × 10^–1^ ng/g, dm) in P4. All four pesticides were detected and quantified in M1, M4, M14 and M15. Overall, M11 had the greatest amount of pesticide (ΣPesticides = 8.1 × 10^3^ ng/g, dm), followed by M14 (ΣPesticides = 1.7 × 10^3^ ng/g, dm) and M6 (ΣPesticides = 1.5 × 10^3^ ng/g, dm). Maize 12 had the lowest pesticide load (ΣPesticides = 9.4 × 10^0^ ng/g, dm) (Table 7). Thus, it can be concluded that selected current-use pesticides are present in the bioavailable fraction of agricultural soils in South Africa.

**Table 7:** Concentrations (ng/g, dm) of pesticides quantified in soils

Sampling location	2,4-D			Atrazine			Dicamba			Imidacloprid			ΣPesticides		
*Mpumalanga province*															
M1	**0.5 × 10**^1^	** ± **	**4.8 × 10**^0^	2.1 × 10^1^	±	6.6 × 10^0^	6.6 × 10^1^	±	2.4 × 10^1^	*9.7 × 10*^1^	* ± *	*3.5 × 10*^1^	1.9 × 10^2^	±	7.1 × 10^1^
M2	7.5 × 10^2^	±	2.2 × 10^2^	4.3 × 10^1^	±	9.8 × 10^0^	**4.9 × 10**^1^	** ±**	**1.8 × 10**^1^	< LOQ			8.4 × 10^2^	±	2.4 × 10^2^
M3	5.5 × 10^2^	±	2.1 × 10^2^	< LOQ			3.5 × 10^2^	±	3.4 × 10^2^	< LOD			9.1 × 10^2^	±	5.5 × 10^2^
M4	2.0 × 10^1^	±	3.1 × 10^0^	2.5 × 10^1^	±	9.6 × 10^0^	1.3 × 10^2^	±	1.2 × 10^2^	5.0 × 10^1^	±	1.5 × 10^1^	2.2 × 10^2^	±	1.5 × 10^2^
M5	< LOQ			4.0 × 10^10^	±	9.0 × 10^–1^	5.4 × 10^2^	±	3.1 × 10^2^	< LOQ			5.5 × 10^2^	±	3.1 × 10^2^
M6	*8.3 × 10*^2^	* ± *	*3.5 × 10*^2^	7.0 × 10^–1^	±	1.0 × 10^–1^	6.3 × 10^2^	±	2.8 × 10^2^	< LOD			1.5 × 10^3^	±	6.3 × 10^2^
M7	< LOD			1.0 × 10^0^	±	2.0 × 10^–1^	4.5 × 10^2^	±	5.0 × 10^2^	< LOQ			4.5 × 10^2^	±	5.0 × 10^2^
M8	5.7 × 10^2^	±	7.5 × 10^2^	*2.1 × 10*^2^	* ± *	*7.8 × 10*^0^	< LOD			< LOQ			7.8 × 10^2^	±	7.6 × 10^2^
M9	5.8 × 10^2^	±	6.0 × 10^2^	8.3 × 10^0^	±	2.3 × 10^0^	7.0 × 10^1^	±	1.2 × 10^2^	< LOQ			6.6 × 10^2^	±	7.2 × 10^2^
M10	< LOQ			1.3 × 10^1^	±	5.6 × 10^0^	< LOD			**2.0 × 10**^1^	** ±**	**5.4 × 10**^0^	3.3 × 10^1^	±	1.1 × 10^1^
M11	< LOQ			3.9 × 10^0^	±	5.8 × 10^0^	*8.1 × 10*^3^	* ± *	*2.6 × 10*^3^	< LOQ			*8.1 × 10*^3^	* ± *	*2.6 × 10*^3^
*Vaalharts Valley, Northern Cape province*															
M12	0.9 × 10^1^	±	5.5 × 10^0^	< LOQ			< LOQ			< LOD			**9.4 × 10**^0^	** ±**	**5.5 × 10**^0^
M13	< LOQ			1.8 × 10^0^	±	1.3 × 10^0^	2.8 × 10^2^	±	2.7 × 10^2^	3.9 × 10^1^	±	2.7 × 10^1^	3.2 × 10^2^	±	3.0 × 10^2^
M14	6.9 × 10^2^	±	5.5 × 10^2^	1.6 × 10^2^	±	8.6 × 10^1^	8.1 × 10^2^	±	7.0 × 10^2^	3.4 × 10^1^	±	1.7 × 10^1^	1.7 × 10^3^	±	1.4 × 10^3^
M15	1.0 × 10^1^	±	1.6 × 10^1^	6.2 × 10^1^	±	2.9 × 10^1^	2.1 × 10^2^	±	2.0 × 10^2^	4.2 × 10^1^	±	2.0 × 10^1^	3.2 × 10^2^	±	2.6 × 10^2^
P1	1.0 × 10^1^	±	3.5 × 10^0^	4.0 × 10^–1^	±	4 × 10^–1^	1.1 × 10^2^	±	1.9 × 10^2^	< LOD			1.2 × 10^2^	±	1.9 × 10^2^
P2	2.2 × 10^1^	±	5.8 × 10^0^	1.3 × 10^0^	±	3.0 × 10^–1^	1.7 × 10^2^	±	1.5 × 10^2^	< LOQ			1.9 × 10^2^	±	1.6 × 10^2^
P3	1.6 × 10^1^	±	1.9 × 10^0^	8.0 × 10^–1^	±	0.8 × 10^–1^	2.5 × 10^2^	±	2.2 × 10^2^	< LOD			2.6 × 10^2^	±	2.2 × 10^2^
P4	1.6 × 10^1^	±	4.7 × 10^0^	**2.0 × 10**^–1^	** ± **	**1.0 × 10**^–1^	1.8 × 10^2^	±	1.7 × 10^2^	< LOQ			1.9 × 10^2^	±	1.8 × 10^2^

Data are presented as the mean concentrations ± standard deviations; Σ: sum; 2,4-D: 2,4-dichlorophenoxyacetic acid; dm: dry mass; LOD: limit of detection; LOQ: limit of quantification; M1–15: maize field 1–15; P1–4: pecan orchard 1–4; values in bold and italic indicate the lowest and greatest concentrations quantified, respectively, for individual pesticides

## Discussion

The goal of this study was to quantify pesticides in and evaluate in vitro effects of water-soluble fractions of agricultural soils in South Africa. The main findings of the research show i) quantifiable levels of 2,4-D, atrazine, dicamba and imidacloprid are present in the water-soluble fraction of agricultural soils from South Africa and ii) all extracts of soil induced oxidative stress, with several samples causing moderate to severe cytotoxicity and/or anti-androgenic effects.

### Pesticides Present in Soil Samples

All four pesticides were detected at quantifiable concentrations in the soil samples, with atrazine detected most often. This was expected since most of the sampling locations were maize fields, and in South Africa, atrazine is extensively applied to maize crops (Dabrowski 2015b). However, some of the atrazine concentrations quantified were greater than those reported in the literature. The maximum concentration of atrazine reported in the current study, 208.6 ng/g (M8), was three- and four-fold greater compared to findings by Wang and Liu (2020) for which Liaoning in China and Riedo et al. (2021) for Switzerland, respectively. On the contrary, Degrendele et al. (2022) reported lesser atrazine concentrations in soils from two agricultural regions in the Western Cape of South Africa. Atrazine concentrations were 8.6 to 22.6 pg/g and 15.5 pg/g for the Hex River Valley and Grabouw, respectively. The lesser atrazine concentrations reported might be because of the type of crops cultivated in the sampling areas. Atrazine is mainly applied to maize, while pome fruits and table grapes are cultivated in Grabouw and the Hex River Valley (Curchod et al. 2020).

Although dicamba had the second-greatest frequency of detection, no comparable studies were found in the literature. Dicamba is volatile in the environment, and volatilisation represents an important exposure route from the site of application towards non-target areas (Oseland et al. 2020). Due to this, excessive use of dicamba has been restricted in several states of the USA (US EPA 2023). However, dicamba is still applied to crops in South Africa and the levels detected in this study show evidence of soil contamination.

The pre- and post-emergent herbicide, 2,4-D, was detected in more than half of the samples. This was anticipated as approximately 60% of the 2,4-D used in South Africa is applied to maize crops (Dabrowski et al. 2014), likely accounting for the herbicide’s high detection frequency. However, the polarity of 2,4-D makes it prone to run-off or leach through soil, with an estimated 92% of the herbicide ending up in water bodies (Mountassif et al. 2008). Although 2,4-D has been reported to be present in South African aquatic environments (Horn et al. 2019), no studies have quantified it in soil. This is probably due to the herbicide’s non-persistent nature. In soil, 2,4-D has a half-life of only 5 days and its amine salts dissociate in less than 3 min under most environmental conditions (Wilson et al. 1997).

Imidacloprid was only detected in six samples. Although the insecticide is primarily used as a seed coating, it can also be applied as a soil treatment or foliar spray (Abu Zeid et al. 2019). However, only 11.5% of imidacloprid in South Africa is used for maize cultivation (Dabrowski et al. 2014). Therefore, the country’s low usage of imidacloprid in maize-growing regions could explain its low detection frequency. Furthermore, the imidacloprid concentrations of the current study (2.0 × 10^1^ to 9.7 × 10^1^ ng/g, dm) are mostly lower than or comparable to previous findings. A maximum imidacloprid concentration of 29.7 ng/g in soil from conventional farming practices in Switzerland was found (Humann-Guilleminot et al. 2019), while concentrations in soil from cocoa farms in Ghana ranged from 4.3 to 251.4 ng/g, dm.

There are no soil quality guidelines for pesticides in South Africa. Yet, atrazine exceeded the environmental quality standard of Latvia for atrazine of 0.2 ng/g (Innovative Sustainable Remediation 2017), while imidacloprid exceeded the New Zealand Environmental Protection Agency’s Environmental Exposure limit of 1 ng/g (Pook and Gritcan 2019). The presence of the four pesticides in the water-soluble fraction of agricultural soils in this study highlights the potential of these pesticides to leach into non-target aquatic environments. Despite this, South Africa only has water quality guidelines for two pesticides: atrazine and endosulfan. Although the water quality guideline for atrazine in aquatic ecosystems is 10 μg/L as stipulated by the Department of Water Affairs and Forestry (DWAF 1996), this guideline has not been updated since 1996 and there are also no guidelines for water used for livestock watering or irrigation in the agricultural sector. This is worrisome as 2,4-D, atrazine, dicamba and imidacloprid have been linked to toxicological effects in non-target organisms including steroid hormone receptor antagonism, testicular dysfunction, infertility, oxidative stress, and lipid peroxidation (Wirbisky et al. 2016; Westlund and Yargeau 2017; Abu Zeid et al. 2019; Gaaied et al. 2019; Ferguson et al. 2022).

### Cell Viability as an Indicator of Toxicity

Here we report that most of the soil samples did not cause cytotoxicity to HuTu-80, H4IIE-*luc*, MDA-kb2 or T47D-KB*luc* cells at the concentrations investigated. However, there was evidence that some samples caused moderate to severe cytotoxicity, mostly at the greatest exposure concentration of 250 mg soil equivalents/mL. Findings by other authors also support the observation that compounds in agricultural soils can affect cell viability in vitro. El-Alam et al. (2018) investigated the cytotoxic potential of agricultural soils from the Bekaa Valley in Lebanon using human hepatic (HepG2) and bronchial epithelial (BEAS-2B) cells, and the results showed that the soil organic extracts were cytotoxic towards the bronchial cells, but less so to the human hepatic cell line (only at 218 and 656.1 mg of polluted soil).

Conversely, a significant increase in cell viability above 100% was also seen. These findings agree with Zhang et al. (2018) who reported increased cell viability in Chinese hamster ovary K1 (CHO-K1) cells following exposure to soil extracts from agricultural fields across China. Since these authors used a cell proliferation assay to determine cytotoxicity, they concluded that compounds in the soil promoted cell proliferation (Zhang et al. 2018). However, in the present study, the MTT assay was used to quantify cell viability. Although the initial Mosmann (1983) publication indicates that the MTT assay can be used to measure cell proliferation, it is important to note that tetrazolium-based assays do not measure the number of viable cells or their growth but approximate metabolic activity (Berridge et al. 2005). Therefore, it is more likely that some of the samples in the current study may have contained polar compounds, which stimulated the viable cells by increasing their metabolic activity.

An interesting observation was that none of the samples that caused cytotoxicity in the MDA-kb2 cells during the MTT assay run in parallel with AR agonism affected cell viability the same way in the MTT assay that was run in parallel with AR antagonism. This response could likely be attributed to the background of testosterone (0.283 ng/mL) the cells received which might have stimulated cell growth. For example, Hwang et al. (2011) found that low doses of testosterone (≤ 28.842 ng/mL) increased cell viability and served as a protective agent by reducing oxidative damage in Leydig cells. Alternatively, the water-soluble extracts used during the assessment of AR agonism and antagonism might have contained different mixtures of chemicals with varying concentrations (Archer and Van Wyk 2015) since the soil extracts and samples were prepared fresh before each of the different bioassays.

### Receptor-mediated Effects

Agricultural soils are direct recipients of agrochemicals and can exhibit bioactivity by mimicking the interaction of endogenous hormones with nuclear receptors including the AhR, AR and ER (Warner et al. 2019). However, at the concentrations investigated in this study, none of the samples caused AhR agonism. This is likely due to the absence of classical AhR agonists which are usually, but not always, non-polar, dioxin-like compounds (Cha et al. 2022, 2023).

Although individual androgen agonists can cause additive effects when combined in mixtures (Blake et al. 2010), there were no androgenic effects observed in the present study. This result is consistent with findings reported for various pesticides. Although AR-mediated effects of 200 pesticide-active ingredients including 2,4-D, atrazine and imidacloprid were evaluated in CHO-K1 cells, none of the compounds was AR agonists (Kojima et al. 2004). The lack of androgenic activity for several pesticides, including atrazine, was also reported by Aït-Aïssa et al. (2010). Based on this, the mechanism of action of the polar compounds present in the soil could be AR antagonism rather than agonism. This is expected as environmental compounds are more likely to interfere with the AR through anti-androgenicity than androgenicity (Conley et al. 2017). Masking effects could also explain the absence of AR agonism. According to Kim et al. (2006), most environmental anti-androgens antagonise the action of androgens via competition with the AR or by reducing the transcriptional activation of target genes. Several authors have reported that anti-androgens mask the effects of androgens during in vitro bioassays (Weiss et al. 2009; Urbatzka et al. 2007; Alvarez-Muñoz et al. 2015).

Since 42% of samples showed anti-androgenic effects, the possibility of AR antagonists masking the effect of weaker androgens is plausible. This conclusion is consistent with the findings of Li et al. (2015) where 54% of soil samples from an agricultural area surrounding the Second Songhua River in China induced AR antagonism. Interestingly, M8 which showed the most AR antagonistic activity (lowest FC value) had the greatest atrazine concentration (2.1 × 10^2^ ng/g, dm). Moreover, all MP samples with AR antagonism contained atrazine. Several endocrine disruptive effects have been reported for atrazine, including the inhibition of testosterone production in the Leydig cells of male rats (Friedmann 2002) and reproductive dysfunction in adult zebrafish (*Danio rerio*) (Wirbisky et al. 2016). However, since the responses for AR antagonism cannot be attributed to individual pesticides with absolute certainty in this study, the samples most likely contained a mixture of AR antagonists which acted jointly on the AR when combined at low concentrations causing the observed anti-androgenic effects (Ma et al. 2019).

Even though environmental xenoestrogens can bind to the ER with a 1000-fold lower affinity compared to endogenous oestrogens (De Falco et al. 2015), none of the samples in the current study caused oestrogenic or anti-oestrogenic effects. Contrary to our study, previous research evidenced that compounds in soil samples interfered with the ER. Li et al. (2015) reported (anti-)oestrogenic effects of soil samples collected along the Second Sanghua River in China, while the Zhang et al. (2018) study showed that 16% and 79% of soil samples caused α-ER antagonistic and agonistic effects in CHO-K1 cells, respectively. The absence of (anti-)oestrogenic effects in the present study, apart from our adaptation to the assay, might be because there were no ER ligands in the samples. Moreover, the response of environmental mixtures in cell-based bioassays is minimal, so because of this, environmental samples are usually concentrated. However, in this study, to mimic environmental conditions as much as possible, extracts of soils were purposefully not concentrated. Concentrated samples might have elicited a response as in the case of Lundqvist et al. (2019).

### Oxidative Stress Responses

In the present study, all samples caused a redox imbalance in HuTu-80 and H4IIE-*luc* cells due to ROS production and/or changes in the level of antioxidant enzymes (i.e., SOD and CAT). This is worrisome as any change in the oxidative balance within cells can alter cell integrity and cause oxidative damage to macromolecules including DNA, lipids and proteins, eventually leading to cell death (Gomez et al. 2020).

The ROS in HuTu-80 cells induced by several samples (M1–M5 and P1) was comparable to or greater than the PC-stimulated cells. However, due to limited cell-based literature about the oxidative stress responses of agricultural soils, these findings could not be compared to previous research. Despite this, other studies have reported increased ROS production following pesticide exposure in vitro. Gargouri et al. (2020) reported a concentration-dependent increase in ROS after human neuroblastoma (SK-N-SH) cells were exposed to the insecticide bifenthrin, while 24 h of acetamiprid exposure significantly increased ROS levels in first-trimester trophoblast cells (HTR-8/SVneo) (Gomez et al. 2020).

Reduction in ROS (M6–M11 and P3) indicates that the antioxidant defence system of the HuTu-80 cells was able to neutralise the ROS and consequently SOD and CAT returned to a balanced state. The reduction of ROS can also increase the antioxidant enzymes as in the case M7, M10 and M11. This suggests SOD was elevated during the conversion of O_2_^•−^ into H_2_O_2_, reducing ROS. CAT activities then increased in response to the generated H_2_O_2_ (Farombi et al. 2008; Basopo and Muzvidziwa 2020). These results are consistent with previous findings that exposure to environmental pollutants usually increases SOD and CAT activity (Farombi et al. 2008). However, prolonged exposures to xenobiotics can reduce SOD while increasing CAT, as with the HuTu-80 cells exposed to M8 and P3. Superoxide dismutase activity may have been inhibited (Ventura et al. 2015), but excess H_2_O_2_ was still present and catalysed by CAT, increasing enzymatic activity (Chance et al. 1948). Contrary to this, the slight increase in SOD content following exposure to M1 and M3 suggests that the enzyme did not entirely detoxify the O_2_^•–^ produced by the cells. Although P1 exhibited similar ROS and CAT responses to M1–M5, there was a significant decrease in SOD content. Superoxide dismutase initially converted O_2_^•–^ into H_2_O_2_ and O_2_, but could perhaps no longer mitigate the production of ROS due to the downregulation of *sod* genes in HuTu-80 cells (Bebe and Panemangalore 2003).

Even though M9 decreased ROS, there was no notable change in SOD content, and O_2_^•−^ in the HuTu-80 cells was likely not neutralised. The subsequent decrease in CAT activity can therefore be attributed to the flux of O_2_^•−^ which is known to inhibit CAT activity (El-Demerdash 2011; Ansari and Ansari 2014) by affecting the haem group located in the active site of the enzyme (Astiz et al. 2009). In the case of M12–M15, P2 and P4 only the antioxidant enzymes in the HuTu-80 cells were affected. Decreased SOD content was probably due to the utilisation of the enzyme which generated great levels of H_2_O_2_, but impaired functional groups in the enzyme’s structure or caused depletion in the process (JanakiDevi et al. 2013; Akbel et al. 2018; Huang et al. 2020). Subsequently, CAT increased to neutralise excess H_2_O_2._

Compared to the HuTu-80 cells, the H4IIE-*luc* cells were able to detoxify the xenobiotics present in the samples to a greater extent with fewer samples causing oxidative stress. The cell lines used in this study originate from separate organ tissues and have distinctive physiological competencies and different basal levels of ROS, SOD and CAT (Ventura et al. 2015). In the current study, the response in the liver cells was expected. The liver is the primary target for chemical-induced toxicity (Tabernilla et al. 2021) because it is located downstream of the GIT and is exposed to the greatest concentrations of pollutants from the oral route (Escher et al. 2021). The lesser responses of the H4IIE-*luc* cells may be due to the liver’s primary function, detoxification. Therefore, the liver’s antioxidant defence system is more robust compared to other organs (Astiz et al. 2009). A four times greater concentration of H_2_O_2_ was necessary to induce ROS in HuTu-80 PC cells compared to H4IIE-*luc* PC cells. This demonstrates that liver cells require significant intoxication before anti-oxidative effects are initiated.

Slightly more samples induced ROS production in the H4IIE-*luc* cells compared to those that reduced it. Maize 1, M13 and P2 increased ROS levels that overwhelmed the antioxidant defence system, preventing any SOD and/or CAT response (Huang et al. 2020). Similar responses of unchanged SOD and CAT activities were reported by Ventura et al. (2015) after two human breast cancer cell lines, MCF-7 and MDA-MB-231, were exposed to the insecticide, chlorpyrifos (0.05 µM; 24 h). Like M1 and M3 in the HuTu-80 cells, M5 and M7 increased ROS and SOD content in the H4IIE-*luc* cells, but did not affect CAT activity. If the conversion of ROS into H_2_O_2_ and O_2_ by SOD was still in progress, intra-cellular H_2_O_2_ would be low and explain why CAT activity remained unchanged. This is supported by Bebe and Panemangalore (2003) who investigated the effect of endosulfan and chlorpyrifos on the endogenous antioxidants in the liver, lungs and erythrocytes of rats. Interestingly, two samples (M12 and M14) induced ROS production but also increased CAT activity, possibly due to the presence of excess H_2_O_2_ in the H4IIE-*luc* cells. Similarly to exposure in the HuTu-80 cells, ROS production was unaffected by some samples suggesting rapid detoxification. Although this was also true for M11, the rapid or continuous conversion of O_2_^•−^ into H_2_O_2_ eventually depleted SOD in the H4IIE-*luc* cells (Kapoor et al. 2010).

Overall, there was no observable trend between the biological effects caused by certain samples and the pesticides quantified in these samples (Supplementary Information, Table S7). However, there was a slight difference in effects and pesticides quantified between the MP and VH samples (Supplementary Information, Table S7). In general, more samples from MP affected cell viability (100%) and caused AR antagonism (55%) compared to the VH samples (50% affected cell viability and 25% caused AR antagonism). The greatest concentration for each of the four pesticides quantified was in an MP sample (Supplementary Information, Table S7). This was expected because the MP is more industrialised compared to the VH. Sampling locations in the MP are surrounded by large-scale agricultural activities and have a large input of pesticides. The MP is also heavily impacted by extensive mining activities that have been ongoing for more than 100 years (Vermeulen and Usher 2009; Simpson et al. 2019). Although the extensive application of agrochemicals might be responsible for the MP’s biological effects, the influences of mining activities cannot be ruled out. Apart from land-use activities, rainfall and the type of irrigation practices followed are other contributing factors. Yet, since the amount of rainfall for the MP and water used for irrigation in the VH during the sampling period is unknown, no definite conclusions can be made.

## Conclusions

The authors have demonstrated that agricultural soils from South Africa contain quantifiable levels of 2,4-D, atrazine, dicamba and imidacloprid and that the water-soluble fraction of these soils causes moderate to severe cytotoxicity, shows anti-androgenic effects and induces oxidative stress in vitro. Consequently, this poses a potential health risk to humans and wildlife.

Moreover, this study shows that cell-based bioassays are a powerful tool for assessing toxicological endpoints of environmental matrices which contain a mixture of chemicals present at low concentrations. However, the identity and concentration of the responsible chemicals cannot be elucidated by bioassays alone. The combined use of instrumental chemical analysis and cell-based bioassay provides a more holistic overview of the effect of agriculture on non-target organisms in the environment.

A matrix-matched calibration curve was used to account for matrix effects the overall pesticide recoveries were low, and the authors recommend that the extraction and quantification methods should be optimised for individual pesticides. Moreover, none of the observed biological effects can directly be attributed to the presence of the four quantified pesticides and it is recommended that the biological effects of 2,4-D, atrazine, dicamba and imidacloprid be evaluated in vitro. Exposures should include pure compounds (i.e., active ingredients), commercial formulations and pesticide mixtures to reflect environmental conditions. Despite these limitations, the findings of this study provide insights into the presence and toxicology of polar compounds in environmental samples.

## Supplementary Information

Below is the link to the electronic supplementary material.

Supplementary file1 (DOCX 1081 KB)

## Data Availability

Data that support the findings of this study are not openly available, but are available from the corresponding author, Ilzé Engelbrecht, upon reasonable request.
