# Author Correction: Quantification of Pesticides and In Vitro Effects of Water-Soluble Fractions of Agricultural Soils in South Africa

**DOI:** 10.1007/s00244-025-01168-z

**Published:** 2025-11-25

**Authors:** Ilzé Engelbrecht, Suranie R. Horn, John P. Giesy, Rialet Pieters

**Affiliations:** 1 Unit for Environmental Sciences and Management, North-West University, Potchefstroom Campus, Private Bag X6001, Potchefstroom, 2520 South Africa; 2 Occupational Hygiene and Health Research Initiative, North-West University, Potchefstroom, 2520 South Africa; 3 Toxicology Program Faculty, Toxicology Centre, University of Saskatchewan, 44 Campus Drive, Saskatoon, SK S7N 5B3 Canada; 4 Department of Veterinary Biomedical Sciences, University of Saskatchewan, Saskatoon, SK S7N 5B4 Canada; 5 Department of Integrative Biology and Center for Integrative Toxicology, Michigan State University, East Lansing, MI 48824 USA; 6 Department of Environmental Science, Baylor University, One Bear Place #97266, Waco, TX 76798‑7266 USA

**Correction to: Archives of Environmental Contamination and Toxicology (2025) 88:230–250** 10.1007/s00244-025-01115-y

**Table 7:** Concentrations (ng/g, dm) of pesticides quantified in soils

Sampling location	2,4-D	Atrazine	Dicamba	Imidacloprid	ΣPesticides
*Mpumalanga province*					
M1	**0.5 x 10** ^**1**^ ** ± 4.8 x 10** ^**0**^	2.1 x 10^1^ ± 6.6 x 10^0^	6.6 x 10^1^ ± 2.4 x 10^1^	*9.7 x 10* ^*1*^ * ± 3.5 x 10* ^*1*^	1.9 x 10^2^ ± 7.1 x 10^1^
M2	7.5 x 10^2^ ± 2.2 x10^2^	4.3 x 10^1^ ± 9.8 x 10^0^	**4.9 x 10** ^**1**^ ** ± 1.8 x 10** ^**1**^	<LOQ	8.4 x 10^2^ ± 2.4 x 10^2^
M3	5.5 x 10^2^ ± 2.1 x10^2^	< LOQ	3.5 x 10^2^ ± 3.4 x 10^2^	<LOD	9.1 x 10^2^ ± 5.5 x 10^2^
M4	2.0 x 10^1^ ± 3.1 x 10^0^	2.5 x 10^1^ ± 9.6 x 10^0^	1.3 x 10^2^ ± 1.2 x 10^2^	5.0 x 10^1^ ± 1.5 x 10^1^	2.2 x 10^2^ ± 1.5 x 10^2^
M5	<LOQ	4.0 x 10^0^ ± 9.0 x 10^-1^	5.4 x 10^2^ ± 3.1 x 10^2^	<LOQ	5.5 x 10^2^ ± 3.1 x 10^2^
M6	*8.3 x 10* ^*2*^ * ± 3.5 x10* ^*2*^	7.0 x 10^-1^ ± 1.0 x 10^-1^	6.3 x 10^2^ ± 2.8 x 10^2^	<LOD	1.5 x 10^3^ ± 6.3 x 10^2^
M7	<LOD	1.0 x 10^0^ ± 2.0 x 10^-1^	4.5 x 10^2^ ± 5.0 x 10^2^	<LOQ	4.5 x 10^2^ ± 5.0 x 10^2^
M8	5.7 x 10^2^ ± 7.5 x 10^2^	*2.1 x 10* ^*2*^ * ± 7.8 x 10* ^*0*^	<LOD	<LOQ	7.8 x 10^2^ ± 7.6 x 10^2^
M9	5.8 x 10^2^ ± 6.0 x 10^2^	8.3 x 10^0^ ± 2.3x 10^0^	7.0 x 10^1^ ± 1.2 x 10^2^	<LOQ	6.6 x 10^2^ ± 7.2 x 10^2^
M10	<LOQ	1.3 x 10^1^ ± 5.6 x 10^0^	<LOD	**2.0 x 10** ^**1**^ ** ± 5.4 x 10** ^**0**^	3.3 x 10^1^ ± 1.1 x 10^1^
M11	< LOQ	3.9 x 10^0^ ± 5.8 x 10^0^	*8.1 x 10* ^*3*^ * ± 2.6 x 10* ^*3*^	<LOQ	*8.1 x 10* ^*3*^ * ± 2.6 x 10* ^*3*^
*Vaalharts Valley, Northern Cape province*					
M12	0.9 x 10^1^ ± 5.5 x 10^0^	<LOQ	<LOQ	<LOD	**9.4 x 10** ^**0**^ ** ± 5.5 x 10** ^**0**^
M13	<LOQ	1.8 x 10^0^ ± 1.3 x 10^0^	2.8 x 10^2^ ± 2.7 x 10^2^	3.9 x 10^1^ ± 2.7 x 10^1^	3.2 x 10^2^ ± 3.0 x 10^2^
M14	6.9 x 10^2^ ± 5.5 x 10^2^	1.6 x 10^2^ ± 8.6 x 10^1^	8.1 x 10^2^ ± 7.0 x 10^2^	3.4 x 10^1^ ± 1.7 x 10^1^	1.7 x 10^3^ ± 1.4 x 10^3^
M15	1.0 x 10^1^ ± 1.6 x 10^1^	6.2 x 10^1^ ± 2.9 x 10^1^	2.1 x 10^2^ ± 2.0 x 10^2^	4.2 x 10^1^ ± 2.0 x 10^1^	3.2 x 10^2^ ± 2.6 x 10^2^
P1	1.0 x 10^1^ ± 3.5 x 10^0^	4.0 x 10^-1^ ± 4 x 10^-1^	1.1 x 10^2^ ± 1.9 x 10^2^	<LOD	1.2 x 10^2^ ± 1.9 x 10^2^
P2	2.2 x 10^1^ ± 5.8 x 10^0^	1.3 x 10^0^ ± 3.0 x 10^-1^	1.7 x 10^2^ ± 1.5 x 10^2^	<LOQ	1.9 x 10^2^ ± 1.6 x 10^2^
P3	1.6 x 10^1^ ± 1.9 x 10^0^	8.0 x 10^-1^ ± 0.8 x 10^-1^	2.5 x 10^2^ ± 2.2 x 10^2^	<LOD	2.6 x 10^2^ ± 2.2 x 10^2^
P4	1.6 x 10^1^ ± 4.7 x 10^0^	**2.0 x 10** ^**-1**^ ** ± 1.0 x 10** ^**-1**^	1.8 x 10^2^ ± 1.7 x 10^2^	<LOQ	1.9 x 10^2^ ± 1.8 x 10^2^

Data are presented as the mean concentrations ± standard deviations; Σ: sum; 2,4−D: 2,4−dichlorophenoxyacetic acid; dm: dry mass; LOD: limit of detection; LOQ: limit of quantification; M1–15: Maize field 1–15; P1–4: Pecan orchard 1–4; values in bold and italic indicate the lowest and greatest concentrations quantified, respectively, for individual pesticides

The atrazine concentration quantified for M5 was incorrectly reported in the initially published article as 4.0 × 10^10^ ± 9.0 × 10^–1^ ng/g, dm, which translates to 40 000 000 000 ng/g, dm. The correct concentration should be 4.0 × 10^0^ ± 9.0 × 10^–1^ ng/g, dm, which translates to only 4 ng/g, dm.The error has been corrected in Table 7 below. The typo of ten to the power of ten (10^10^) instead of ten to the power of zero (10^0^) significantly impacts the reported concentration. This error only appears once in the published article (in Table 7) and did not affect the interpretation of the results or conclusions made.

