# Supplementary material for: Quantification of Pesticides and In Vitro Effects of Water-Soluble Fractions of Agricultural Soils in South Africa
Source: Arch Environ Contam Toxicol. 2025 Feb 15;88(2):230–50. doi: 10.1007/s00244-025-01115-y (PMC11870950; doi:10.1007/s00244-025-01115-y)
Supplement: Supplementary file 1 — Supplementary file1 (DOCX 1081 KB) [file 244_2025_1115_MOESM1_ESM.docx]

**Supplementary Information**

*Archives of Environmental Contamination and Toxicology*

Quantification of pesticides and *in vitro* effects of water-soluble fractions of agricultural soils in South Africa

Ilzé Engelbrecht^*^, Suranie R. Horn, John P. Giesy, Rialet Pieters

*Corresponding author; Unit for Environmental Sciences and Management, North-West University, Potchefstroom, 2520, South Africa; Occupational Hygiene and Health Research Initiative, North-West University, Potchefstroom, 2520, South Africa; [24997803@mynwu.ac.za](mailto:24997803@mynwu.ac.za)

**Materials and methods**

**Cell viability assay (MTT)** At the start of the assay, cells were seeded in their respective nutrient medium (Table 1) in 96-well, clear, flat-bottom microplates. Following 24 hours of adherent cell growth, the initial nutrient media of the cells were removed and replaced with the samples (28, 83 and 250 mg soil equivalents/mL). For the MTT assay only the greatest three sample exposure concentrations were used. Cells treated with a solvent control (SC) and negative control (NC, i.e., methanol-killed cells) were also included. After 24 (HuTu-80 and T47D-KB*luc*), 48 (MDA-kb2) and 72 hours (H4IIE-*luc*) of exposure, the cells were washed with DPBS and incubated with freshly prepared 0.5 mg/mL MTT solution for 2 hours. The formazan crystals were dissolved in 200 µL DMSO and the absorbance was quantified spectrophotometrically at 560 nm using a multimode microplate reader. The results were expressed as a percentage (%) of the SC which represents 100% viable cells. All exposures were done in triplicate.

**Xenobiotic metabolism** During the H4IIE‑*luc* bioassay, cells were seeded in a hormone-stripped medium (Table 1) in 96-well, clear flat-bottom, white-walled microplates and incubated for 24 hours. Following attachment, the cells were exposed to the samples by replacing the nutrient media with 250 µL of the samples (i.e., media replacement). The reference compound was 2,3,7,8-tetrachlorodibenzo-*para*-dioxin (TCDD) serially diluted in hexane (0.06, 0.37, 2.2, 13.3, 80 and 480 pg/mL). After 72 hours of exposure, the nutrient media was removed and the cells were washed three times with DPBS (containing Ca^2+^ and Mg^2+^ as CaCl_2_ and MgSO_4_.7H_2_O, respectively), followed by the addition of 25 µL reporter lysis buffer and rapid freezing at -80°C. Following one freeze‑thaw cycle, luminescence was quantified with a multimode microplate reader which automatically injects 100 µL of a luciferase assay reagent [20 mM tricine, 1.07 mM (MgCO_3_)_4_Mg(OH)_2_.5H_2_O), 2.67 mM MgSO_4_.7H_2_O, 0.1 mM EDTA, 33.3 mM DTT, 270 µM coenzyme A, 530 µM ATP, and 470 µM beetle luciferin potassium salt] (Villeneuve et al. 1999).

**(Anti-)androgenic activity** During the assessment of receptor agonism, cells were seeded in hormone-stripped medium in 96-well microplates (Table 1). Following 48 hours of incubation the cells were exposed to the samples. Testosterone serially diluted in methanol (8, 47, 142, 283, 567 and 850 pg/mL) served as the reference compound for AR agonism. After 48 hours of exposure, luciferase activity was determined as previously described. Since the MDA-kb cells contain both the AR and GR, agonism could indicate ligand binding to either of these receptors. To distinguish whether compounds in the samples bind to the AR or GR, flutamide (a known AR antagonist) was co-administered during a second bioassay. In this case, cells were seeded as described above but with a background concentration of flutamide (0.5942 µg/mL) (Table 1). The cells were exposed to the samples for 48 hours, while dexamethasone serially diluted in methanol (0.55, 2.20, 8.79, 35.16, 140.63, and 562.50 ng/mL) was the reference compound. If any receptor binding occurred, it was attributed to the presence of GR agonists. Evaluating receptor antagonism, on the other hand, requires the presence of a constant concentration of a known agonist (Escher et al. 2021). For AR antagonism, the cells were seeded in in hormone-stripped medium in 96-well microplates with a background concentration of testosterone (0.283 ng/mL) (Table 1). This was done to ensure 80% activation of the AR (Escher et al. 2021). After 48 hours of incubation, the cells were exposed to the samples. Flutamide serially diluted in methanol (0.02, 0.06, 0.19, 0.56, 1.67 and 5.0 µg/mL) was the reference compound. Following 48 hours of exposure, luciferase activity was determined.

**ROS generation** The cells were seeded in 24-well microplates and incubated for 24 hours (Table 1). Following attachment, the cells were exposed to the greatest non-toxic sample concentration (i.e., 83 mg/mL) through media replacement for 24 hours. Untreated cells were included as the control (C), while HuTu-80 and H4IIE-*luc* cells stimulated with 3.5 ng/mL and 14.2 ng/mL H_2_O_2_, respectively, for 45 minutes served as the ROS positive control (PC). These concentrations were based on previous optimisation experiments (results not shown) since the H_2_O_2_ concentration required to induce ROS varies between cell lines due to their different antioxidative capabilities. After exposure, the cells were washed with DPBS, and H_2_DCF-DA (10 µM) was added to the wells, followed by incubation for 30 minutes. To harvest the cell content, the cells were trypsinised and transferred to a microcentrifuge tube for centrifugation at 1 000 g and 25°C for 4 minutes. The supernatant was discarded, and the cell pellet re-suspended in DPBS. Cell fluorescence was measured at excitation and emission wavelengths of 480 nm and 535 nm, respectively, using a multi-mode microplate reader. Results were compared to the controls.

**Protein determination and antioxidant enzyme assays** For SOD content, CAT activity and protein determination, the cells were treated in the same manner as described above for ROS generation. However, after the cells had been harvested, the cell pellet was re-suspended in an ice‑cold potassium phosphate buffer (0.09 M) and cells lysed through ultrasonication (medium intensity, 30 seconds) and centrifugation (10 000 g, 4 minutes, and 4°C). This supernatant was used to determine protein and SOD content, as well as CAT activity. For protein determination, 5 µL supernatant was transferred to a 96-well, clear-bottom microplate, followed by the addition of 245 µL Bradford’s reagent. The absorbance was measured spectrophotometrically at 590 nm and the protein content of the cells was calculated using a BSA (0–2 000 µg/mL) standard curve.For SOD content, 4 µL supernatant was transferred to a clear-bottom 96-well microplate, followed by the addition of 245 µL DTPA/tris-buffer (1:49) solution containing 1 nM DTPA and 50 mM tris-buffer. The reaction was initiated by adding 4 µL pyrogallol (24 nM in 10 mM HCl acidified deionised water). Absorbance was measured spectrophotometrically at 560 nm every 30 seconds for 4.5 minutes. The reaction rate was calculated and expressed as ng SOD/mg protein (Marklund and Marklund 1974; Del Maestro and McDonald 1987). For CAT activity, 4 µL supernatant aliquot was added to a clear-bottom 96-well microplate. The reaction was initiated by the addition of 93 µL H_2_O_2_ (6 mM), followed by incubation at 37°C for 3 minutes. Sulphuric acid (6M) was used to stop the reaction and the amount of H_2_O_2_ remaining was determined by the addition of 130 µL KMnO_4_ (1.9 mM). The residual KMnO_4_ was determined spectrophotometrically by immediately measuring absorbance at 490 nm. A CAT blank comprised of only reagents and without cells was included. First-order kinetics describe the enzyme‑catalysed decomposition of H_2_O_2_ by CAT and was used to calculate the enzymatic activity. Obtained values were normalised against protein content and expressed as µM H_2_O_2_/min/mg protein (Cohen et al. 1970; Mennillo et al. 2019). During the oxidative stress bioassays all exposures were done in triplicate.

**Extraction for instrumental analysis** For the extraction, 100 mL stainless steel extraction cells were used. Methanol was the extraction solvent and the parameters for the DIONEX ASE 100^®^ were set to 60˚C, 10 342 kPa, 10 minutes heat with 5 minutes static, 60% flush volume, nitrogen purge time of 100 seconds, and three cycles. The crude extract was collected, and the extracts were evaporated until dryness with nitrogen gas. The samples were reconstituted in 1 mL methanol containing 1% formic acid and passed through a 0.22 µm Acrodisc^®^ prefilter to remove any particulates before chemical analysis.

**Quantification of target pesticides** Chemical analysis of the soil samples was performed using an ultra-high performance liquid chromatography coupled to quadrupole time-of-flight mass spectrometry (UHPLC-QTOF/MS) system comprised of an Agilent 1290 Infinity binary pump, autosampler and thermostatted column compartment; coupled to an Agilent 6540 Accurate Mass QTOF/M. Briefly, 1 µL of the soil extract was injected into an Agilent ZORBAX Eclipse Plus C18 Rapid Resolution HD (2.1 mm x 50 mm, 1.8 µm particle size) column for the separation of peaks. The column temperature was maintained at 60°C and the binary pump flow rate was 0.3 mL/minute. The mass axis of the Q-TOF was calibrated for positive and negative ionisation states daily using the Agilent tuning mix. The mobile phases and gradients used to ensure optimum chromatographic separation are listed in Table S1. The drying gas temperature was set to 200°C, with a gas flow of 9 L/min and nebuliser pressure of 276 kPa. Sheath gas temperature and gas flow were 250°C and 10 L/min, respectively. The following voltage specifications were used: capillary voltage of 3 500 V; nozzle voltage of 1 000 V; fragmentor voltage of 175 V; skimmer voltage of 35 V; and octopole RF peak to peak voltage of 750 V. The instrument was set to scan from 100 to 3 000 m/z and was operated in the extended dynamic range mode (2 GHz) at 1 spectra/second. The total run time was 22 and 10 minutes per run for positive and negative modes, respectively.

**Table S1** The gradient used for the mobile phases during positive and negative electrospray ionisation (ESI) methods

| **Gradient (min)** | **Mobile phase A (%)**  **Ultrapure water + 0.1% formic acid** | **Mobile phase B (%)**  **Acetonitrile + 0.1% formic acid** |
| --- | --- | --- |
| *Positive ESI* | | |
| 4.00 | 85.00 | 15.00 |
| 4.10 | 75.00 | 25.00 |
| 12.00 | 75.00 | 25.00 |
| 12.10 | 55.00 | 45.00 |
| 15.00 | 55.00 | 45.00 |
| 15.10 | 0.00 | 100.00 |
| 20.00 | 0.00 | 100.00 |
| 20.10 | 85.00 | 15.00 |
| 22.00 | 85.00 | 15.00 |
| *Negative ESI* | | |
| 2.00 | 95.00 | 5.00 |
| 2.10 | 70.00 | 30.00 |
| 6.00 | 70.00 | 30.00 |
| 6.10 | 0.00 | 100.00 |
| 7.00 | 0.00 | 100.00 |
| 7.10 | 95.00 | 5.00 |

**Results**

**Fig. S1** Dose-response curve for aryl hydrocarbon receptor agonism reference compound, 2,3,7,8-tetrachlorodibenzo-p-dioxin (TCDD), during the H4IIE-*luc* bioassay. Luciferase activity is expressed as %TCDD Max against the logarithmically transformed TCDD exposure concentrations (0.06, 0.37, 2.2, 13.3, 80, and 480 pg/mL). Error bars indicate standard deviation.


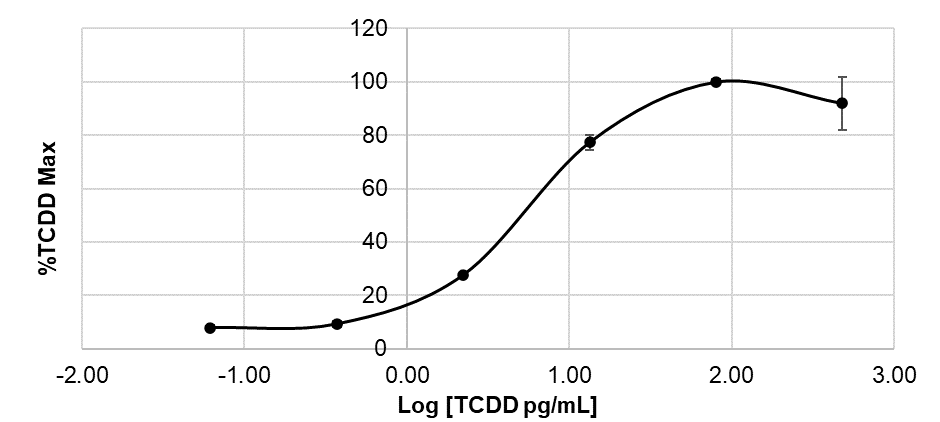


**Table S2** %TCDD Max values of the H4IIE-*luc* cells after exposure to the samples during the assessment of aryl hydrocarbon receptor agonism

| Sampling location | Exposure concentration (mg/mL) | %TCDD Max | | |
| --- | --- | --- | --- | --- |
| BC | N/A | *13 | ± | 10.03 |
| SC | N/A | 6 | ± | 1.79 |
| *Mpumalanga province* | | | | |
| M1 | 1 | 6 | ± | 1.06 |
|  | 3 | 8 | ± | 1.36 |
|  | 9 | 5 | ± | 1.19 |
|  | 28 | 6 | ± | 1.08 |
|  | 83 | 5 | ± | 1.26 |
|  | 250 | 5 | ± | 1.70 |
| M2 | 1 | 6 | ± | 1.04 |
|  | 3 | 6 | ± | 1.87 |
|  | 9 | 6 | ± | 1.79 |
|  | 28 | 5 | ± | 2.34 |
|  | 83 | 5 | ± | 1.11 |
|  | 250 | *1 | ± | 0.95 |
| M3 | 1 | 5 | ± | 0.76 |
|  | 3 | 6 | ± | 0.89 |
|  | 9 | 6 | ± | 0.67 |
|  | 28 | 6 | ± | 0.08 |
|  | 83 | 5 | ± | 2.20 |
|  | 250 | 6 | ± | 0.36 |
| M4 | 1 | 6 | ± | 0.29 |
|  | 3 | 6 | ± | 0.50 |
|  | 9 | 7 | ± | 0.71 |
|  | 28 | 7 | ± | 0.22 |
|  | 83 | 8 | ± | 0.33 |
|  | 250 | 7 | ± | 0.63 |
| M5 | 1 | 6 | ± | 0.14 |
|  | 3 | 6 | ± | 0.23 |
|  | 9 | 7 | ± | 1.09 |
|  | 28 | 7 | ± | 0.80 |
|  | 83 | 7 | ± | 0.43 |
|  | 250 | 6 | ± | 0.53 |
| M6 | 1 | 7 | ± | 0.13 |
|  | 3 | 8 | ± | 0.50 |
|  | 9 | 8 | ± | 0.37 |
|  | 28 | *8 | ± | 0.58 |
|  | 83 | 8 | ± | 1.24 |
|  | 250 | 4 | ± | 3.21 |
| M7 | 1 | 4 | ± | 0.51 |
|  | 3 | 5 | ± | 0.35 |
|  | 9 | 5 | ± | 1.28 |
|  | 28 | *4 | ± | 0.56 |
|  | 83 | *3 | ± | 0.62 |
|  | 250 | *1 | ± | 0.41 |
| M8 | 1 | *3 | ± | 0.44 |
|  | 3 | 5 | ± | 1.07 |
|  | 9 | 6 | ± | 2.45 |
|  | 28 | *9 | ± | 1.53 |
|  | 83 | 5 | ± | 2.54 |
|  | 250 | *2 | ± | 0.77 |
| M9 | 1 | *2 | ± | 0.41 |
|  | 3 | *3 | ± | 0.33 |
|  | 9 | 5 | ± | 0.86 |
|  | 28 | 6 | ± | 1.07 |
|  | 83 | 8 | ± | 1.73 |
|  | 250 | *10 | ± | 0.20 |
| M10 | 1 | 4 | ± | 1.59 |
|  | 3 | 5 | ± | 1.32 |
|  | 9 | 5 | ± | 1.34 |
|  | 28 | 6 | ± | 1.65 |
|  | 83 | 7 | ± | 2.50 |
|  | 250 | 6 | ± | 4.73 |
| *Vaalharts Valley, Northern Cape province* | | | | |
| M11 | 1 | 5 | ± | 0.62 |
|  | 3 | 7 | ± | 0.51 |
|  | 9 | 7 | ± | 0.95 |
|  | 28 | *8 | ± | 0.71 |
|  | 83 | *9 | ± | 1.53 |
|  | 250 | 6 | ± | 1.17 |
| M12 | 1 | 6 | ± | 1.37 |
|  | 3 | 6 | ± | 0.89 |
|  | 9 | 7 | ± | 1.99 |
|  | 28 | *8 | ± | 0.91 |
|  | 83 | 8 | ± | 0.92 |
|  | 250 | *4 | ± | 1.13 |
| M13 | 1 | 7 | ± | 0.67 |
|  | 3 | 7 | ± | 0.29 |
|  | 9 | 8 | ± | 0.07 |
|  | 28 | 7 | ± | 1.43 |
|  | 83 | *2 | ± | 3.05 |
|  | 250 | 3 | ± | 2.88 |
| M14 | 1 | *3 | ± | 0.29 |
|  | 3 | 5 | ± | 0.31 |
|  | 9 | 4 | ± | 0.99 |
|  | 28 | *2 | ± | 0.99 |
|  | 83 | *3 | ± | 0.44 |
|  | 250 | *0 | ± | 0.03 |
| M15 | 1 | 7 | ± | 1.18 |
|  | 3 | *8 | ± | 0.65 |
|  | 9 | *8 | ± | 0.43 |
|  | 28 | *11 | ± | 0.65 |
|  | 83 | *9 | ± | 0.55 |
|  | 250 | 6 | ± | 0.47 |
| P1 | 1 | 5 | ± | 0.03 |
|  | 3 | 7 | ± | 0.48 |
|  | 9 | *8 | ± | 0.90 |
|  | 28 | *9 | ± | 0.41 |
|  | 83 | *9 | ± | 0.56 |
|  | 250 | *8 | ± | 0.70 |
| P2 | 1 | 6 | ± | 0.98 |
|  | 3 | 7 | ± | 0.33 |
|  | 9 | 8 | ± | 1.95 |
|  | 28 | *11 | ± | 0.55 |
|  | 83 | 7 | ± | 0.12 |
|  | 250 | 6 | ± | 0.42 |
| P3 | 1 | 8 | ± | 0.70 |
|  | 3 | *8 | ± | 0.92 |
|  | 9 | *8 | ± | 0.24 |
|  | 28 | *9 | ± | 0.64 |
|  | 83 | 8 | ± | 0.33 |
|  | 250 | 5 | ± | 0.98 |
| P4 | 1 | *8 | ± | 0.32 |
|  | 3 | *8 | ± | 0.35 |
|  | 9 | *8 | ± | 0.36 |
|  | 28 | *9 | ± | 0.28 |
|  | 83 | *9 | ± | 0.56 |
|  | 250 | 6 | ± | 0.10 |

Data is presented as mean ± standard deviation. *Statistically significant compared to the SC (*p* ≤ 0.05). BC: blank control; M1–M15: Maize field 1–Maize field 15; N/A: not applicable; P1–P4: Pecan orchard 1–Pecan orchard 4; SC: solvent control

**Fig. S2** Dose-response curve for androgen receptor agonism reference compound, testosterone, during the MDA-kb2 bioassay. Luciferase activity is expressed as %Testosterone Max against the logarithmically transformed testosterone exposure concentrations (8, 47, 142, 283, 567, and 850 pg/mL). Error bars indicate the standard deviation


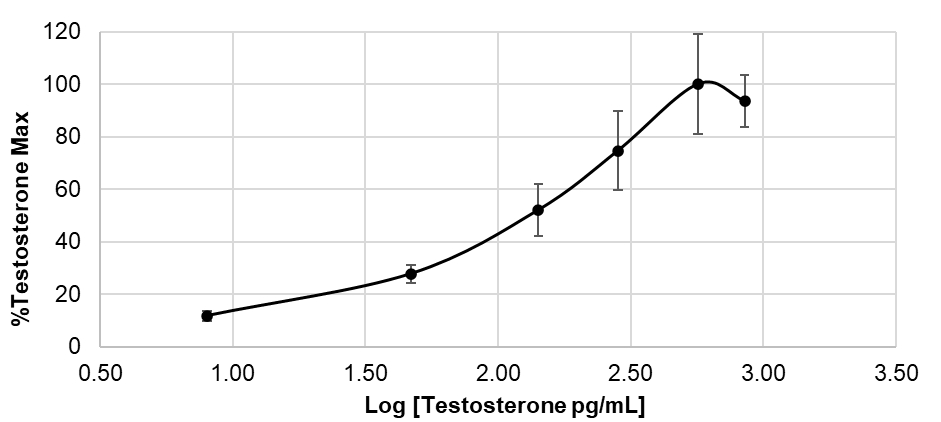


**Table S3** %Testosterone Max values of the MDA-kb2 cells after exposure to the samples during the assessment of androgen receptor agonism

| Sampling location | Exposure concentration (mg/mL) | %Testosterone Max | | |
| --- | --- | --- | --- | --- |
| BC | N/A | *12 | ± | 2.42 |
| SC | N/A | 11 | ± | 2.49 |
| *Mpumalanga province* | | | | |
| M1 | 1 | *18 | ± | 1.79 |
|  | 3 | *13 | ± | 0.42 |
|  | 9 | *16 | ± | 1.53 |
|  | 28 | *16 | ± | 5.28 |
|  | 83 | 14 | ± | 8.53 |
|  | 250 | *3 | ± | 0.15 |
| M2 | 1 | *17 | ± | 1.84 |
|  | 3 | *14 | ± | 2.12 |
|  | 9 | *14 | ± | 0.25 |
|  | 28 | *14 | ± | 0.72 |
|  | 83 | 11 | ± | 1.28 |
|  | 250 | 10 | ± | 0.17 |
| M3 | 1 | 11 | ± | 1.15 |
|  | 3 | 11 | ± | 1.17 |
|  | 9 | 11 | ± | 0.48 |
|  | 28 | 12 | ± | 1.82 |
|  | 83 | 12 | ± | 2.08 |
|  | 250 | 11 | ± | 1.09 |
| M4 | 1 | 12 | ± | 1.54 |
|  | 3 | 12 | ± | 0.81 |
|  | 9 | *13 | ± | 0.28 |
|  | 28 | 12 | ± | 1.78 |
|  | 83 | 12 | ± | 1.73 |
|  | 250 | 12 | ± | 0.75 |
| M5 | 1 | 12 | ± | 1.59 |
|  | 3 | 12 | ± | 0.34 |
|  | 9 | 11 | ± | 2.01 |
|  | 28 | 11 | ± | 0.99 |
|  | 83 | 10 | ± | 1.99 |
|  | 250 | *8 | ± | 0.38 |
| M6 | 1 | 10 | ± | 1.09 |
|  | 3 | 11 | ± | 0.67 |
|  | 9 | 10 | ± | 1.22 |
|  | 28 | 11 | ± | 2.79 |
|  | 83 | *7 | ± | 2.09 |
|  | 250 | *3 | ± | 0.51 |
| M7 | 1 | *14 | ± | 0.65 |
|  | 3 | 13 | ± | 0.89 |
|  | 9 | 13 | ± | 3.69 |
|  | 28 | *17 | ± | 3.27 |
|  | 83 | *14 | ± | 0.39 |
|  | 250 | 12 | ± | 3.41 |
| M8 | 1 | 13 | ± | 2.66 |
|  | 3 | 13 | ± | 2.97 |
|  | 9 | 12 | ± | 1.59 |
|  | 28 | *14 | ± | 2.13 |
|  | 83 | *15 | ± | 0.83 |
|  | 250 | *15 | ± | 1.66 |
| M9 | 1 | 11 | ± | 1.15 |
|  | 3 | 13 | ± | 1.35 |
|  | 9 | 12 | ± | 4.24 |
|  | 28 | 9 | ± | 4.46 |
|  | 83 | 13 | ± | 2.85 |
|  | 250 | 10 | ± | 3.27 |
| M10 | 1 | 12 | ± | 0.94 |
|  | 3 | 12 | ± | 1.00 |
|  | 9 | 12 | ± | 2.24 |
|  | 28 | *16 | ± | 0.96 |
|  | 83 | 10 | ± | 1.01 |
|  | 250 | *14 | ± | 1.90 |
| M11 | 1 | 12 | ± | 0.57 |
|  | 3 | 11 | ± | 0.48 |
|  | 9 | 13 | ± | 0.54 |
|  | 28 | *13 | ± | 0.49 |
|  | 83 | 8 | ± | 5.89 |
|  | 250 | *5 | ± | 1.02 |
| *Vaalharts Valley, Northern Cape province* | | | | |
| M12 | 1 | 10 | ± | 0.84 |
|  | 3 | 10 | ± | 0.58 |
|  | 9 | 10 | ± | 0.57 |
|  | 28 | 10 | ± | 0.72 |
|  | 83 | 9 | ± | 1.96 |
|  | 250 | 9 | ± | 2.07 |
| M13 | 1 | 16 | ± | 5.39 |
|  | 3 | *16 | ± | 1.50 |
|  | 9 | *17 | ± | 1.82 |
|  | 28 | *14 | ± | 2.51 |
|  | 83 | 13 | ± | 0.36 |
|  | 250 | 8 | ± | 3.51 |
| M14 | 1 | *19 | ± | 3.04 |
|  | 3 | *17 | ± | 2.02 |
|  | 9 | *18 | ± | 1.14 |
|  | 28 | *17 | ± | 1.69 |
|  | 83 | 12 | ± | 2.85 |
|  | 250 | 9 | ± | 2.87 |
| M15 | 1 | 11 | ± | 0.90 |
|  | 3 | 12 | ± | 0.80 |
|  | 9 | *14 | ± | 0.94 |
|  | 28 | 13 | ± | 2.18 |
|  | 83 | *17 | ± | 0.59 |
|  | 250 | 9 | ± | 3.70 |
| P1 | 1 | 11 | ± | 0.83 |
|  | 3 | 13 | ± | 0.83 |
|  | 9 | 11 | ± | 0.92 |
|  | 28 | 11 | ± | 0.84 |
|  | 83 | 11 | ± | 1.71 |
|  | 250 | *8 | ± | 0.93 |
| P2 | 1 | 11 | ± | 2.64 |
|  | 3 | 13 | ± | 2.01 |
|  | 9 | 10 | ± | 1.28 |
|  | 28 | 13 | ± | 1.11 |
|  | 83 | 12 | ± | 1.36 |
|  | 250 | *3 | ± | 4.43 |
| P3 | 1 | 10 | ± | 1.36 |
|  | 3 | 13 | ± | 1.34 |
|  | 9 | 12 | ± | 0.91 |
|  | 28 | *16 | ± | 4.03 |
|  | 83 | 9 | ± | 3.29 |
|  | 250 | *4 | ± | 0.58 |
| P4 | 1 | *8 | ± | 0.74 |
|  | 3 | *8 | ± | 1.03 |
|  | 9 | *8 | ± | 0.92 |
|  | 28 | *16 | ± | 2.18 |
|  | 83 | *16 | ± | 1.02 |
|  | 250 | 14 | ± | 3.17 |

Data is presented as mean ± standard deviation. *Statistically significant compared to the SC (*p* ≤ 0.05). BC: blank control; M1–M15: Maize field 1–Maize field 15; N/A: not applicable; P1–P4: Pecan orchard 1–Pecan orchard 4; SC: solvent control

**Fig. S3** Cell viability (%) of the MDA-kb2 (AR antagonism) cells after exposure to samples. Results are expressed in terms of the solvent control (SC) that represents 100% viability (dashed line). All data is presented as mean ± standard deviation. *Statistically significant compared to SC (*p* ≤ 0.05); M1–15: Maize field 1–15; NC: negative control; P1–4: Pecan orchard 1–4

**Fig. S4** Dose-response curve for oestrogen receptor agonism reference compound, 17β-oestradiol (E_2_), during the T47D-KB*luc* bioassay. Luciferase activity is expressed as %E_2_ Max against the logarithmically transformed E_2_ exposure concentrations (0.02, 0.11, 0.39, 1.36, 2.72, and 6.81 pg/mL). Error bars indicate the standard deviation


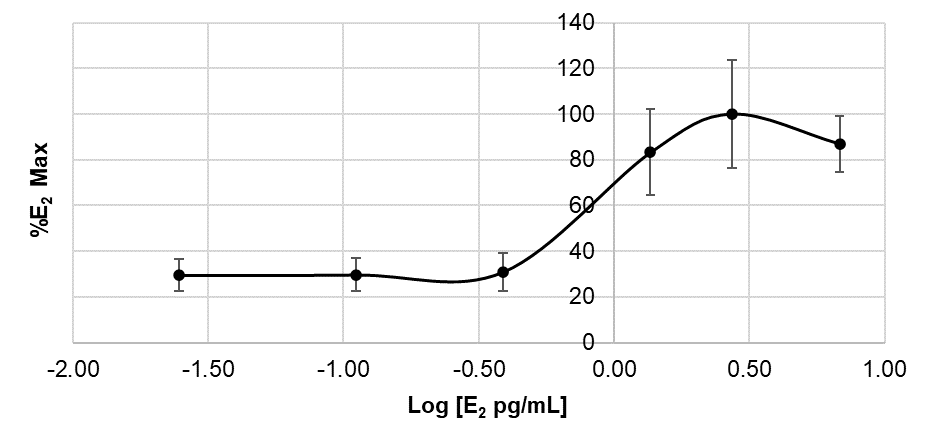


**Table S4** %E_2_ Max values of the T47D-KB*luc* cells after exposure to the samples during the assessment of oestrogen receptor agonism

| Sampling location | Exposure concentration (mg/mL) | %E_2_ Max | | |
| --- | --- | --- | --- | --- |
| BC | N/A | 28 | ± | 8.71 |
| SC | N/A | 27 | ± | 9.76 |
| *Mpumalanga province* | | | | |
| M1 | 1 | 25 | ± | 3.35 |
|  | 3 | 21 | ± | 2.62 |
|  | 9 | 32 | ± | 13.51 |
|  | 28 | 22 | ± | 5.24 |
|  | 83 | *16 | ± | 1.52 |
|  | 250 | *3 | ± | 0.28 |
| M2 | 1 | 20 | ± | 2.89 |
|  | 3 | 20 | ± | 1.39 |
|  | 9 | 20 | ± | 3.05 |
|  | 28 | 26 | ± | 4.00 |
|  | 83 | *18 | ± | 0.79 |
|  | 250 | *6 | ± | 0.72 |
| M3 | 1 | 21 | ± | 1.68 |
|  | 3 | *18 | ± | 1.25 |
|  | 9 | *16 | ± | 1.63 |
|  | 28 | *16 | ± | 0.85 |
|  | 83 | *12 | ± | 1.26 |
|  | 250 | *7 | ± | 1.01 |
| M4 | 1 | *15 | ± | 1.56 |
|  | 3 | *16 | ± | 1.97 |
|  | 9 | *15 | ± | 1.58 |
|  | 28 | *13 | ± | 1.32 |
|  | 83 | *9 | ± | 0.85 |
|  | 250 | *5 | ± | 0.87 |
| M5 | 1 | *14 | ± | 1.47 |
|  | 3 | *13 | ± | 1.00 |
|  | 9 | *13 | ± | 0.07 |
|  | 28 | *12 | ± | 0.72 |
|  | 83 | *10 | ± | 1.03 |
|  | 250 | *7 | ± | 0.43 |
| M6 | 1 | *15 | ± | 0.64 |
|  | 3 | *18 | ± | 0.98 |
|  | 9 | *15 | ± | 0.76 |
|  | 28 | *19 | ± | 1.34 |
|  | 83 | *13 | ± | 0.41 |
|  | 250 | *8 | ± | 0.25 |
| M7 | 1 | *19 | ± | 0.64 |
|  | 3 | *17 | ± | 0.22 |
|  | 9 | *17 | ± | 0.85 |
|  | 28 | *16 | ± | 0.56 |
|  | 83 | *16 | ± | 1.61 |
|  | 250 | *7 | ± | 1.32 |
| M8 | 1 | *17 | ± | 1.04 |
|  | 3 | *17 | ± | 1.18 |
|  | 9 | *18 | ± | 1.58 |
|  | 28 | *18 | ± | 2.14 |
|  | 83 | *13 | ± | 1.02 |
|  | 250 | *8 | ± | 1.30 |
| M9 | 1 | 22 | ± | 1.48 |
|  | 3 | 24 | ± | 2.39 |
|  | 9 | 22 | ± | 1.17 |
|  | 28 | *17 | ± | 0.30 |
|  | 83 | *13 | ± | 0.31 |
|  | 250 | *10 | ± | 0.65 |
| M10 | 1 | 22 | ± | 1.48 |
|  | 3 | 24 | ± | 2.39 |
|  | 9 | 22 | ± | 1.17 |
|  | 28 | *17 | ± | 0.30 |
|  | 83 | *13 | ± | 0.31 |
|  | 250 | *10 | ± | 0.65 |
| M11 | 1 | 22 | ± | 0.72 |
|  | 3 | 22 | ± | 0.83 |
|  | 9 | 22 | ± | 2.53 |
|  | 28 | 22 | ± | 2.60 |
|  | 83 | 23 | ± | 0.78 |
|  | 250 | *18 | ± | 0.68 |
| *Vaalharts Valley, Northern Cape province* | | | | |
| M12 | 1 | *18 | ± | 3.28 |
|  | 3 | *19 | ± | 2.13 |
|  | 9 | *19 | ± | 1.08 |
|  | 28 | 27 | ± | 1.22 |
|  | 83 | 34 | ± | 1.82 |
|  | 250 | *8 | ± | 0.52 |
| M13 | 1 | *16 | ± | 1.08 |
|  | 3 | *15 | ± | 1.19 |
|  | 9 | *14 | ± | 1.31 |
|  | 28 | *48 | ± | 3.25 |
|  | 83 | *15 | ± | 3.75 |
|  | 250 | *8 | ± | 1.58 |
| M14 | 1 | *16 | ± | 1.46 |
|  | 3 | *17 | ± | 0.97 |
|  | 9 | *15 | ± | 3.10 |
|  | 28 | 19 | ± | 3.60 |
|  | 83 | *18 | ± | 1.94 |
|  | 250 | *17 | ± | 2.51 |
| M15 | 1 | 29 | ± | 3.91 |
|  | 3 | 30 | ± | 0.78 |
|  | 9 | 30 | ± | 1.30 |
|  | 28 | 29 | ± | 1.70 |
|  | 83 | *20 | ± | 1.48 |
|  | 250 | *8 | ± | 0.42 |
| P1 | 1 | 24 | ± | 4.45 |
|  | 3 | 29 | ± | 2.73 |
|  | 9 | 31 | ± | 3.64 |
|  | 28 | 28 | ± | 3.64 |
|  | 83 | 24 | ± | 2.76 |
|  | 250 | *17 | ± | 3.38 |
| P2 | 1 | 26 | ± | 2.88 |
|  | 3 | *41 | ± | 2.71 |
|  | 9 | 34 | ± | 7.35 |
|  | 28 | 29 | ± | 3.59 |
|  | 83 | 25 | ± | 3.58 |
|  | 250 | *14 | ± | 1.41 |
| P3 | 1 | 24 | ± | 2.26 |
|  | 3 | 26 | ± | 2.47 |
|  | 9 | 31 | ± | 1.95 |
|  | 28 | 34 | ± | 2.89 |
|  | 83 | *38 | ± | 2.94 |
|  | 250 | *38 | ± | 0.49 |
| P4 | 1 | *11 | ± | 1.04 |
|  | 3 | *13 | ± | 2.78 |
|  | 9 | *19 | ± | 0.57 |
|  | 28 | *17 | ± | 1.71 |
|  | 83 | *16 | ± | 1.14 |
|  | 250 | *10 | ± | 1.16 |

Data is presented as mean ± standard deviation. *Statistically significant compared to the SC (*p* ≤ 0.05). BC: blank control; M1–M15: Maize field 1–Maize field 15; N/A: not applicable; P1–P4: Pecan orchard 1–Pecan orchard 4; SC: solvent contro

**Fig. S5** Dose-response curve for the oestrogen receptor antagonism reference compound, ICI, during the T47D-KB*luc* bioassay. Luciferase activity is expressed as %ICI Max against the logarithmically transformed ICI exposure concentrations (0.02, 0.05, 0.16, 0.47, 1.41, and 4.22 ng/mL). Error bars indicate the standard deviation


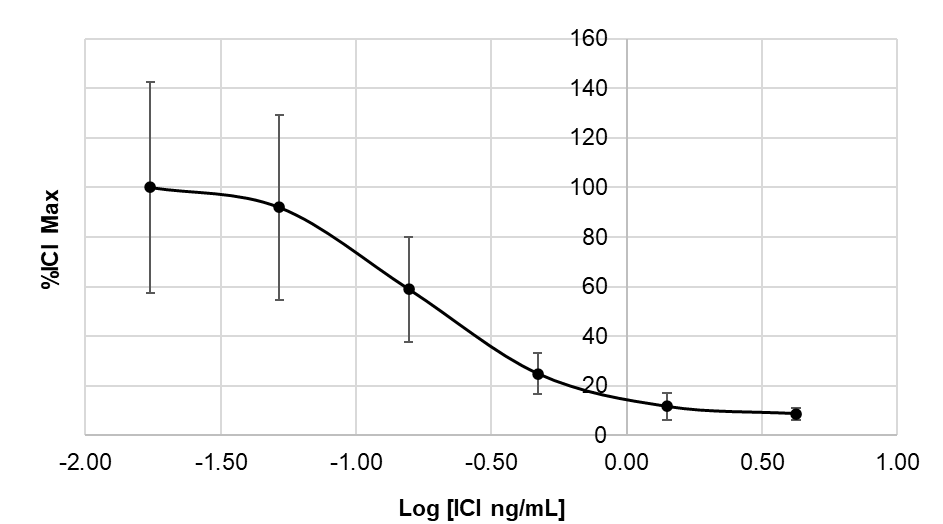


**Table S5** %ICI Max values of the T47D-KB*luc* cells after exposure to the samples during the assessment of oestrogen receptor antagonism

| Sampling location | Exposure concentration (mg/mL) | %ICI Max | | |
| --- | --- | --- | --- | --- |
| BC | N/A | 103 | ± | 32.37 |
| SC | N/A | 109 | ± | 39.93 |
| *Mpumalanga province* | | | | |
| M1 | 1 | 136 | ± | 4.31 |
|  | 3 | 176 | ± | 3.72 |
|  | 9 | 112 | ± | 23.50 |
|  | 28 | 118 | ± | 11.06 |
|  | 83 | 144 | ± | 41.45 |
|  | 250 | 161 | ± | 47.58 |
| M2 | 1 | 194 | ± | 8.36 |
|  | 3 | 99 | ± | 10.78 |
|  | 9 | 94 | ± | 8.30 |
|  | 28 | 92 | ± | 11.70 |
|  | 83 | 118 | ± | 17.36 |
|  | 250 | *212 | ± | 21.10 |
| M3 | 1 | 121 | ± | 8.03 |
|  | 3 | 114 | ± | 33.87 |
|  | 9 | 222 | ± | 18.55 |
|  | 28 | 100 | ± | 30.75 |
|  | 83 | 94 | ± | 18.49 |
|  | 250 | *137 | ± | 9.23 |
| M4 | 1 | 90 | ± | 6.47 |
|  | 3 | 123 | ± | 18.11 |
|  | 9 | 111 | ± | 39.51 |
|  | 28 | 73 | ± | 1.49 |
|  | 83 | 72 | ± | 10.25 |
|  | 250 | *198 | ± | 27.38 |
| M5 | 1 | 126 | ± | 13.39 |
|  | 3 | 113 | ± | 2.55 |
|  | 9 | 96 | ± | 6.86 |
|  | 28 | 140 | ± | 10.26 |
|  | 83 | 100 | ± | 3.88 |
|  | 250 | 141 | ± | 39.39 |
| M6 | 1 | *141 | ± | 8.95 |
|  | 3 | 122 | ± | 2.26 |
|  | 9 | 146 | ± | 42.41 |
|  | 28 | 122 | ± | 16.83 |
|  | 83 | 127 | ± | 3.71 |
|  | 250 | *186 | ± | 32.10 |
| M7 | 1 | 89 | ± | 6.76 |
|  | 3 | 118 | ± | 13.94 |
|  | 9 | 126 | ± | 22.39 |
|  | 28 | 106 | ± | 11.74 |
|  | 83 | 105 | ± | 17.90 |
|  | 250 | *169 | ± | 25.23 |
| M8 | 1 | 99 | ± | 9.26 |
|  | 3 | 120 | ± | 18.42 |
|  | 9 | 94 | ± | 5.25 |
|  | 28 | 113 | ± | 25.17 |
|  | 83 | 107 | ± | 11.83 |
|  | 250 | 100 | ± | 30.14 |
| M9 | 1 | 84 | ± | 14.15 |
|  | 3 | 105 | ± | 27.32 |
|  | 9 | 100 | ± | 26.32 |
|  | 28 | 104 | ± | 15.63 |
|  | 83 | 114 | ± | 28.83 |
|  | 250 | 109 | ± | 26.13 |
| M10 | 1 | 105 | ± | 2.49 |
|  | 3 | 96 | ± | 0.38 |
|  | 9 | 99 | ± | 4.57 |
|  | 28 | 101 | ± | 4.40 |
|  | 83 | 108 | ± | 2.87 |
|  | 250 | *143 | ± | 13.86 |
| M11 | 1 | 107 | ± | 14.65 |
|  | 3 | 94 | ± | 3.68 |
|  | 9 | 91 | ± | 10.52 |
|  | 28 | 97 | ± | 7.41 |
|  | 83 | 95 | ± | 13.33 |
|  | 250 | 126 | ± | 25.10 |
| *Vaalharts Valley, Northern Cape province* | | | | |
| M12 | 1 | 91 | ± | 24.99 |
|  | 3 | 102 | ± | 6.25 |
|  | 9 | 85 | ± | 6.87 |
|  | 28 | 96 | ± | 25.20 |
|  | 83 | 114 | ± | 11.99 |
|  | 250 | 106 | ± | 19.21 |
| M13 | 1 | *61 | ± | 6.38 |
|  | 3 | *57 | ± | 10.57 |
|  | 9 | 75 | ± | 36.97 |
|  | 28 | *51 | ± | 10.59 |
|  | 83 | *44 | ± | 13.66 |
|  | 250 | *58 | ± | 14.30 |
| M14 | 1 | 53 | ± | 4.26 |
|  | 3 | *57 | ± | 12.20 |
|  | 9 | *51 | ± | 6.34 |
|  | 28 | *57 | ± | 4.41 |
|  | 83 | *63 | ± | 7.63 |
|  | 250 | 114 | ± | 35.78 |
| M15 | 1 | 117 | ± | 17.07 |
|  | 3 | *166 | ± | 44.10 |
|  | 9 | *212 | ± | 30.94 |
|  | 28 | 120 | ± | 11.27 |
|  | 83 | 148 | ± | 27.72 |
|  | 250 | *168 | ± | 13.01 |
| P1 | 1 | 92 | ± | 20.46 |
|  | 3 | 124 | ± | 13.76 |
|  | 9 | 125 | ± | 12.08 |
|  | 28 | 132 | ± | 4.16 |
|  | 83 | *152 | ± | 20.86 |
|  | 250 | *146 | ± | 19.14 |
| P2 | 1 | 118 | ± | 35.69 |
|  | 3 | 90 | ± | 13.46 |
|  | 9 | *173 | ± | 31.06 |
|  | 28 | 147 | ± | 44.26 |
|  | 83 | 133 | ± | 55.91 |
|  | 250 | 236 | ± | 57.38 |
| P3 | 1 | 124 | ± | 24.56 |
|  | 3 | 104 | ± | 15.37 |
|  | 9 | 127 | ± | 38.27 |
|  | 28 | 98 | ± | 6.20 |
|  | 83 | 144 | ± | 33.31 |
|  | 250 | *225 | ± | 21.68 |
| P4 | 1 | *58 | ± | 6.84 |
|  | 3 | *56 | ± | 12.76 |
|  | 9 | 75 | ± | 33.02 |
|  | 28 | 72 | ± | 15.60 |
|  | 83 | 79 | ± | 27.46 |
|  | 250 | 125 | ± | 9.27 |

Data is presented as mean ± standard deviation. *Statistically significant compared to the SC (*p* ≤ 0.05). BC: blank control; M1–M15: Maize field 1–Maize field 15; N/A: not applicable; P1–P4: Pecan orchard 1–Pecan orchard 4; SC: solvent control

**Table S6** Oxidative stress responses in the HuTu-80 and H4IIE-*luc* cells after exposure to samples (83 mg soil equivalents/mL) for 24 and 72 hours, respectively

| **Sampling location** | **HuTu-80** | | | | | | | | | **H4IIE-*luc*** | | | | | | | | |
| --- | --- | --- | --- | --- | --- | --- | --- | --- | --- | --- | --- | --- | --- | --- | --- | --- | --- | --- |
|  | **ROS production** | | | **SOD content** | | | **CAT activity** | | | **ROS production** | | | **SOD content** | | | **CAT activity** | | |
|  | **(RFUs)** | | | **(ng SOD/mg protein)** | | | **(µM H_2_O_2_/min/mg protein)** | | | **(RFUs)** | | | **(ng SOD/mg protein)** | | | **(µM H_2_O_2_/min/mg protein)** | | |
| *Mpumalanga province* | | | | | | | | | | | | | | | | | | |
| C | 39 691 | ± | 16 222 | 122.74 | ± | 62.33 | 27.87 | ± | 21.79 | 37 837 | ± | 8 303 | 68.97 | ± | 35.91 | 42.07 | ± | 28.75 |
| PC | **53 617** | **±** | **7 562** |  | - |  |  | - |  | ****69 628** | **±** | **30 366** |  | - |  |  | - |  |
| M1 | ****60 795** | **±** | **8 487** | *160.23 | ± | 11.2 | 17.23 | ± | 3.74 | ****65 713** | **±** | **21 688** | 72.7 | ± | 32.76 | 41.51 | ± | 24.78 |
| M2 | ****74 896** | **±** | **8 265** | 159.22 | ± | 43.57 | 12.43 | ± | 2.55 | 36 349 | ± | 5 110 | ***110.52** | **±** | **66.62** | 44.96 | ± | 17.89 |
| M3 | ****60 658** | **±** | **15 635** | *162.74 | ± | 22.13 | 14.98 | ± | 3.24 | 42 548 | ± | 14 101 | 93.74 | ± | 39.58 | 43 | ± | 22.42 |
| M4 | ***55 513** | **±** | **8 806** | 161.98 | ± | 44.39 | 15.16 | ± | 3.3 | ***30 794** | **±** | **4 190** | **98.85** | **±** | **43.63** | 39 | ± | 8.33 |
| M5 | ****79 877** | **±** | **21 732** | 142.08 | ± | 36.85 | 13.31 | ± | 3.41 | ****54 919** | **±** | **10 508** | ****113.75** | **±** | **54.41** | 35.6 | ± | 14.71 |
| M6 | ****12 862** | **±** | **3 935** | 154.44 | ± | 12.12 | 12.83 | ± | 3.05 | 38 214 | ± | 3 816 | ****118.94** | **±** | **61.19** | 38.62 | ± | 14.282 |
| M7 | ****9 559** | **±** | **1 888** | ***182.14** | **±** | **35.7** | **48.6** | **±** | **19.5** | *42 576 | ± | 4 137 | ****135.91** | **±** | **82.86** | 43.89 | ± | 19.35 |
| M8 | ****11 521** | **±** | **2 203** | ****10.72** | **±** | **8.36** | ****60.60** | **±** | **20.98** | **31 108** | **±** | **8 322** | ****106.68** | **±** | **34.24** | 47.33 | ± | 15.42 |
| M9 | ****13 457** | **±** | **3 990** | 126.72 | ± | 34.66 | *12.13 | ± | 3.57 | **30 415** | **±** | **3 093** | 91.05 | ± | 37.53 | ****73.52** | **±** | **26.74** |
| M10 | ****12 972** | **±** | **4 182** | ***180.04** | **±** | **61.49** | ***44.38** | **±** | **15.97** | 43 517 | ± | 5 361 | 59.46 | ± | 34.26 | 45.22 | ± | 16.79 |
| M11 | ****12 804** | **±** | **1 102** | ****289.33** | **±** | **79.09** | ****62.17** | **±** | **22.33** | 33 301 | ± | 7 487 | ***40.29** | **±** | **37.46** | 37.05 | ± | 32.06 |
| Vaalharts Valley, Northern Cape province | | | | | | | | | | | | | | | | | | |
| M12 | 35 788 | ± | 10 495 | ***62.45** | **±** | **63.62** | ****56.13** | **±** | **13.82** | ****67 580** | **±** | **18 337** | 78.91 | ± | 33.12 | ***64.39** | **±** | **25.98** |
| M13 | 27 671 | ± | 8 847 | ****11.18** | **±** | **7.47** | *43.74 | ± | 7.86 | ***57 962** | **±** | **20 845** | 84.32 | ± | 44.59 | 53.09 | ± | 20.18 |
| M14 | 37 884 | ± | 16 282 | ****48.53** | **±** | **72.49** | ****54.18** | **±** | **15.14** | ****60 888** | **±** | **14 667** | 78.08 | ± | 26.7 | *58.96 | ± | 16.13 |
| M15 | 44 886 | ± | 31 143 | ****44.87** | **±** | **69.86** | ***46.25** | **±** | **21.95** | ****27 261** | **±** | **2 754** | 57.44 | ± | 37.83 | 61.81 | ± | 42.78 |
| P1 | ***60 604** | **±** | **13 934** | ****21.31** | **±** | **15.99** | 32.92 | ± | 12.95 | 33 741 | ± | 9 170 | 71.78 | ± | 25.76 | *59.37 | ± | 21.54 |
| P2 | 42 425 | ± | 16 043 | ****21.09** | **±** | **28.68** | ***47.17** | **±** | **17.99** | **44 134** | **±** | **15 070** | 54.02 | ± | 28.33 | 49.19 | ± | 21.22 |
| P3 | **26 722** | **±** | **8 677** | ****39.60** | **±** | **45.14** | ***50.32** | **±** | **27.9** | ****17 888** | **±** | **3 178** | 66.85 | ± | 24.63 | **63.11 | ± | 18.88 |
| P4 | 36 402 | ± | 10 305 | ****47.27** | **±** | **21.11** | ***46.74** | **±** | **14.22** | ****19 379** | **±** | **4 730** | 57.43 | ± | 42.72 | 39.7 | ± | 32.98 |

Data are presented as the mean ± standard deviation; asterisks indicate statistically significant differences compared to the untreated control (C) (**p* ≤ 0.05 and ***p* ≤ 0.01); values in bold indicate practically significant differences compared to the C (d ≥ 0.8); CAT: catalase; H_2_O_2_: hydrogen peroxide; M1–15: Maize field 1–15; P1–4: Pecan orchard 1–4; PC: positive; RFUs: relative fluorescence units; ROS: reactive oxygen species; SOD: superoxide dismutase

**Table S7** Combined results of the biological effects measured in the four respective cell lines following exposure to the samples and the concentration of target pesticides quantified in the samples

2,4-D: 2,4-Dichlorophenoxyacetic acid; AhR: aryl hydrocarbon receptor; AR: androgen receptor; CAT: catalase activity; dm: dry mass; ER: oestrogen receptor; LOD: limit of detection; LOQ: limit of quantification; M1-15: Maize field 1–15; P1–4: Pecan orchard 1-4; ROS; reactive oxygen species generation; SOD: superoxide dismutase content; -no statistically significant response; 🗴antagonism; 🡡increase; 🡣decrease; <below; values in green and red indicate the lowest and greatest concentrations quantified, respectively, for individual pesticides
